# Global impact of environmental temperature and BCG vaccination coverage on the transmissibility and fatality rate of COVID-19

**DOI:** 10.1371/journal.pone.0240710

**Published:** 2020-10-22

**Authors:** Amit Kumar, Shubham Misra, Vivek Verma, Ramesh K. Vishwakarma, Vineet Kumar Kamal, Manabesh Nath, Kiran Prakash, Ashish Datt Upadhyay, Jitendra Kumar Sahu

**Affiliations:** 1 Department of Neurology, All India Institute of Medical Sciences, New Delhi, India; 2 Department of Biostatistics & Bioinformatics, King Abdullah International Medical Research Center/King Saud bin Abdulaziz University for Health Sciences, Riyadh, Saudi Arabia; 3 Division of Epidemiology & Biostatistics, National Institute of Epidemiology, Indian Council of Medical Research, Chennai, India; 4 Department of Physiology, Government Medical College and Hospital, Chandigarh, India; 5 Department of Biostatistics, All India Institute of Medical Sciences, New Delhi, India; 6 Pediatric Neurology Unit, Postgraduate Institute of Medical Education & Research, Chandigarh, India; University of Surrey, School of Veterinary Medicine, UNITED KINGDOM

## Abstract

The 2019-Coronavirus (COVID-19) pandemic has had a global impact. The effect of environmental temperature on transmissibility and fatality rate of COVID-19 and protective efficacy of Bacillus Calmette-Guérin (BCG) vaccination towards COVID-19 remains ambiguous. Therefore, we explored the global impact of environmental temperature and neonatal BCG vaccination coverage on transmissibility and fatality rate of COVID-19. The COVID-19 data for reported cases, deaths and global temperature were collected from 31^st^ December 2020 to 3^rd^ April 2020 for 67 countries. Temperature data were split into quartiles for all three categories (minimum temperature, maximum temperature and mean temperature). The impact of three types of temperature data and policy of BCG vaccination on COVID-19 infection was determined by applying the multivariable two-level negative binomial regression analysis keeping daily new cases and daily mortality as outcome. The highest number of cases fell in the temperature categories as following: *mean temperature in the second quartile* (6°C to 10.5°C), median 26, interquartile range (IQR) 237; *minimum temperature in the first quartile* (-26°C to 1°C), median 23, IQR 173; *maximum temperature in the second quartile (*10°C to 16°C), median 27.5, IQR 219. *For the minimum temperature category*, 28% statistically significant lower incidence was noted for new cases from the countries falling in the second quartile (2°C to 6°C) compared with countries falling in the first quartile (-26°C to 1°C) (incidence rate ratio [IRR] 0.72, 95% confidence interval [CI] 0.57 to 0.93). However, no statistically significant difference in incidence rate was observed for mean temperature categories in comparison to the first quartile. Countries with BCG vaccination policy had 58% less mortality as compared with countries without BCG coverage (IRR 0.42; 95% CI 0.18 to 0.95). Our exploratory study provides evidence that high temperature might not be associated with low transmissibility and countries having neonatal BCG vaccination policy had a low fatality rate of COVID-19.

## Introduction

In December 2019, a cluster of cases with complaints of pneumonia and respiratory illnesses were reported in Wuhan, China [1]. The infections were investigated to be caused by a novel RNA beta-coronavirus which has been termed as severe acute respiratory syndrome coronavirus-2 (COVID-19). Since then, it has become an international outbreak and has infected more than 16,000,000 people and caused the death of more than 600,000 people as on 27^th^ July, 2020 [2]. Since the novel coronavirus has spread rapidly to multiple countries, the World Health Organization (WHO) declared COVID-19 as a public health emergency and a global pandemic [3]. After its inception last year, the virus has spread across all the continents of the globe except for the Antarctic region [4, 5].

The novel SARS-CoV-2 originates from a large family of Coronaviridae nested under the sub-family of Ortho coronaviruses belonging to the beta-coronavirus attribute that primarily infects mammals and birds [6–8]. It is a positive single-strand RNA coronavirus having a genome of approximately 30kb length which encodes for ten genes, four of which give rise to structural proteins such as spike (S), envelope (E), membrane (M) and nucleocapsid (N) [7, 9–11]. This COVID-19 has structural similarities to the beta-coronavirus that has caused two epidemics in the past 18 years; severe acute respiratory syndrome coronavirus (SARS-CoV) and Middle East respiratory syndrome coronavirus (MERS-CoV) which are also of zoonotic origin [8, 12]. As per the literature available till now, the COVID-19 has been found to be less pathogenic (~5%) than its predecessors, SARS-CoV (10%) and MERS-CoV (40%) despite sharing the same human cell receptor with SARS-CoV. However, the transmissibility of COVID-19 is found to be higher (R0: 2–2.5) than both SARS-CoV (R0: 1.7–1.9) and MERS-CoV (R0: <1), establishing the potentiality of SARS-CoV-2 to cause a global pandemic [13, 14].

The influenza virus spread and transmissibility have been found to be affected by the weather conditions, and it needs to be examined if COVID-19 also has similar effects. The half-life of SARS-CoV-2 fomites living on surfaces indoors was shown to reduce drastically at temperatures higher than 30 degree Celsius (°C) [15]. It has been observed from the daily reported data that spread is more common in the region where the temperature lies between 3 to 18°C. Tropical countries are less affected as compared to countries falling in the temperate region, but the advent of monsoon has seen a rise of infections across tropical countries. One study described the impact of cold weather and the rapid increase of COVID-19 infection, with a gradual reduction in cases during summer but with the possibility of recurrence during the autumn season [16]. Temperatures of 5 to 15°C were reported to be optimal for the growth of SARS-CoV-2 in a global study while fewer infections were observed in colder (<0°C) and warmer (>30°C) regions [17]. The effect of temperature on the severity and mortality of COVID-19 infection as well as its transmissibility has also been recently reported in several studies in the Chinese cities [18–20]. Other studies have also indicated that pathogenicity and transmissibility of COVID-19 may be affected by climatic factors like temperature and humidity [21, 22]. However, large numbers of incidence and mortality have been reported after the publications of these articles and significant association with temperature change depicting the global scenario are warranted.

It has also been observed that differences in the incidences and mortality associated with COVID-19 have been affected by national policies of neonatal Bacillus Calmette–Guérin (BCG) vaccination programs in certain countries [23]. Neonatal BCG vaccination has been observed to generate favorable non-specific immunological response leading to better preparedness against other non-mycobacterium pathogens, including viruses. BCG vaccination has been observed to provide some form of immunity, which indirectly correlates to fewer cases in countries with active vaccination policies [24, 25]. Therefore, it is important to examine whether all-cause mortality associated with COVID-19 outbreak is affected by the neonatal BCG vaccination.

It is imperative to speculate the behaviour of COVID-19 in coming days as the climate will change largely in many countries in the coming months, which may directly affect the incidence and mortality associated with COVID-19 outbreak. The decision-making of governments could be framed for lock-down period of their respective countries if the effect of climatic conditions can be examined with the incidence and mortality rate associated with COVID-19 infection.

Therefore, our study aims to determine the association of temperature and neonatal BCG vaccination coverage of respective countries with the spread of the COVID-19 infection and mortality across various countries around the world.

## Methods

### Sampling strategies

The present study was based on the secondary data extracted from authenticated electronic sources (Worldometers (https://www.worldometers.info/coronavirus/), European Centre for Disease Control (http://www.ecdc.europa.eu/en/publications-data /download-todays-data-geographic-distribution -covid-19-cases-worldwide). The reported data for COVID-19 are regularly updated on standard website on daily basis. We obtained the global temperature data from the online database of National Centers for Environmental Information (NCEI) under the National Oceanic and Atmospheric Administration (NOAA) located in the United States of America (USA) for the period between 31^st^ December 2019 to 3^rd^ April, 2020 (NOAA/NCEI, 2020) [26].

The epidemiological data on the incidence and mortality of COVID-19 cases as reported by public health authorities worldwide was accessed till 03^rd^ April 2020 from the website of Our World in Data (https://ourworldindata.org/coronavirus-source-data). In the present study, criteria for inclusion of countries was those countries (67 countries out of 173 countries), where at least one death due to COVID-19 infection was reported till 03^rd^ April 2020. The geographic distribution for the spread of COVID-19 infection across various countries was also visualized on a weekly basis. Since, a cluster of COVID-19 cases outside China was reported only after 21^st^ January 2020, therefore, the geographic distribution of COVID-19 was visualized worldwide from a period of 22^nd^ January 2020 to 03^rd^ April 2020. The daily reported cases and mortality data of COVID-19 along with the temperature data for various countries were divided on a weekly basis viz., week 1 (22 JAN 2020 to 25 JAN 2020) to week 11 (29 MAR 2020 to 03 APR 2020).

For conducting the multilevel negative binomial regression analysis, the data was extracted from 31^st^ December 2019, when a cluster of cases with COVID-19 infection was officially reported in China till 03^rd^ April 2020, in order to consider all the cases reported thus far to detect the effect of temperature and neonatal BCG vaccination coverage in 67 countries on the daily spread and mortality due to COVID-19 outbreak.

The daily average, minimum and maximum temperature of each country or state, were collected for the city or region with the maximum number of confirmed COVID-19 cases.

For example, in the case of China and the United States of America (USA), the maximum number of reported cases came from Wuhan city and New York City, respectively. As such, temperature data of these regions were downloaded for the defined time period. If the number of confirmed cases were less, then temperature data for the most populous city was recorded as large cities were assumed to be the most affected. Temperature data were split into quartiles for all three categories of temperature (minimum temperature, maximum temperature and mean temperature). The data for BCG vaccination programmes were extracted from the BCG world atlas (www.bcgatlas.org) to investigate whether the different BCG vaccination policies adopted by the countries would have any effect on the transmissibility & mortality related to the COVID-19 infection [27]. The total COVID-19 tests conducted in the countries included in our study were obtained from the online data source for reporting COVID-19 statistics [28].

### Statistical analyses

Continuous data were represented by mean with standard deviation (SD) or median with interquartile range (IQR). We had considered temperature and BCG vaccination as potential predictors for both the outcomes, i.e., number of new cases and mortality per day. Since the outcome variables (number of new cases, mortality counts per day) are count (non-negative discrete number), Poisson regression is used as a standard model for analyzing such data. However, in the case of over dispersion, i.e., variance greater than mean, multilevel negative binomial regression model (NBRM) is recommended [29]. In our case, an over dispersion test was performed to evaluate the adequacy of the NBRM over the Poisson regression model. When the ratio of variance to mean of the Poisson distribution value α (alpha) is larger than one, then it indicates over dispersion.

In this study, the variance was greater than the mean, and the over dispersion test was significant, indicating an over dispersion. Hence, the NBRM was preferred over the Poisson model. We considered the number of days from the first case reported to the last date of study (i.e., 3rd April 2020) as exposure in NBRM. We did not consider zero-inflated NBRM as we assumed that there was no scope for excess zeros. Since this data is generated from different countries; finally, we adopted a multilevel NBRM over the traditional count model (NBRM) considering a country as a cluster. Multilevel modeling explicitly accounts for the clustering of the units of analysis, individuals nested within groups or clusters. The effect of the association was quantified by unadjusted and adjusted incidence rate with its 95% confidence interval (CI) using bivariable and multivariable two-level negative binomial regression, respectively. The number of tests conducted in the respective countries was considered as a potential confounder and adjusted in the multivariable model. All the statistical analyses were performed using STATA software, version 13.0 and SAS software, version 9.4 (SAS Institute Inc, Cary, North Carolina). A P-value of less than 0.05 was considered to indicate statistical significance.

## Results

### Geographical distribution of cases

**Fig 1:**
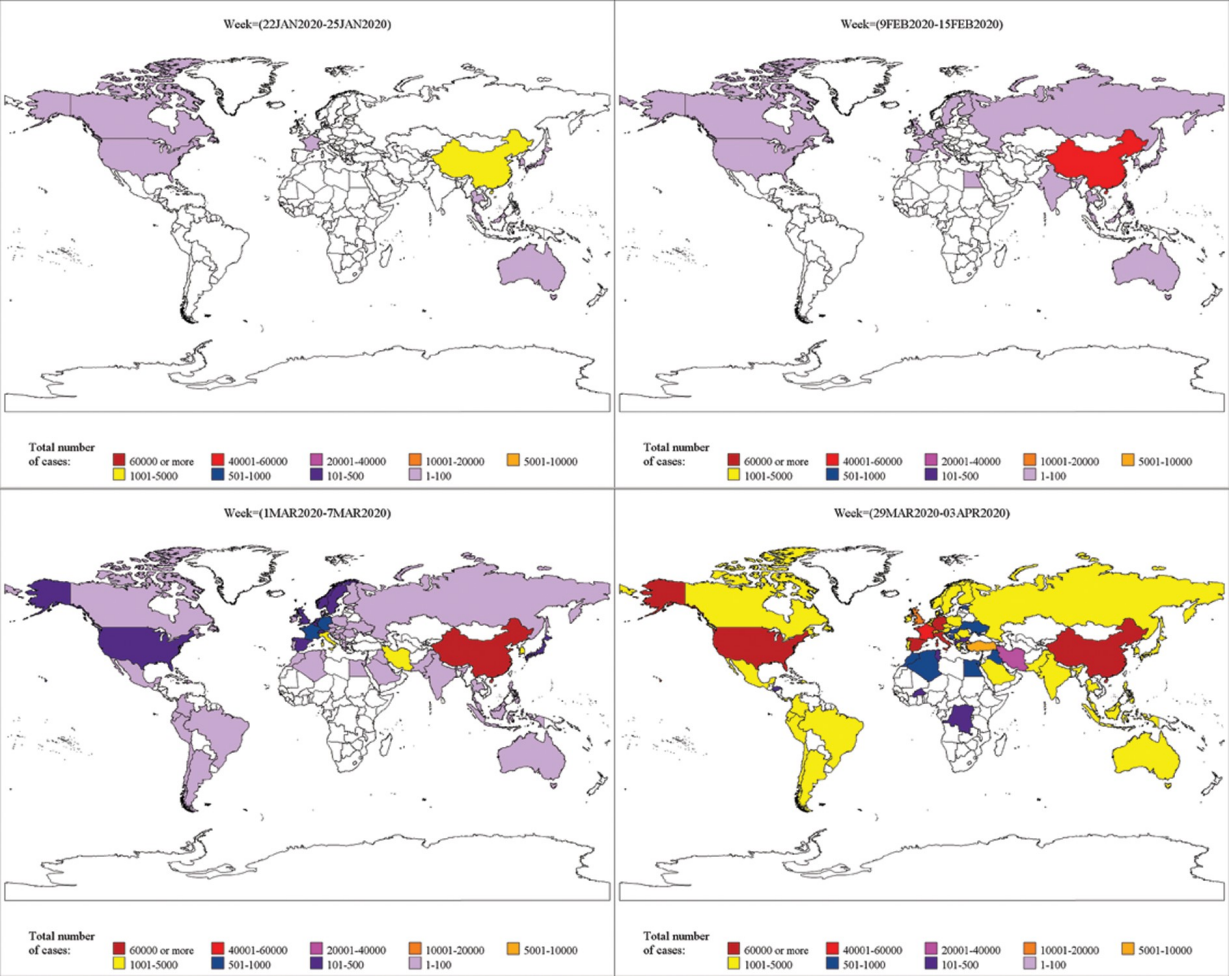
Global transition of infectious cases due to COVID-19 reported in Week 1, Week 4, Week 7 and Week 11.

As of 3^rd^ April 2020, USA reported 2,45,540 confirmed COVID-19 cases followed by Italy (1,15,242), Spain (1,10,238), China (82,465) and Germany (73,522). Whereas, the most casualties related to COVID-19 were reported in Italy 13,917 (12.07%) followed by Spain 10,003 (9.07%) and USA 6,053 (2.47%). The geographic distribution for Week 1, Week 4, Week 7 and Week 11 of the worldwide COVID-19 cases is given in Fig 1. The S1 Fig represents the overall weekly geographic distribution of COVID-19 cases worldwide. For the realization purpose, we have represented total cases of COVID-19 as a spread across nine countries viz., China, Australia, Japan, Malaysia, South Korea, Thailand, France, Canada and the USA wherein the highest confirmed cases were reported from China (3,354). In week 1, the minimum temperature in China was 4°C, whereas maximum temperature was 8°C. Australia and Canada had reported the lowest number of COVID-19 cases in week one and had a minimum temperature of 16°C and 0°C and a maximum temperature of 34°C and -2°C, respectively (S2 Fig). The geographical distribution of cases in week 2 depicted that infection had spread in nine more countries, including India, in addition to the countries reported previously. During the said period, after China, the maximum COVID-19 cases were reported in Thailand and Japan. After the completion of 4 weeks, the infection had spread in more than 38 countries with China being on top followed by South Korea, Italy and Iran. In the highest infected countries with the greatest number of reported cases, the minimum temperature lied between 1°C to 4°C and a maximum temperature was between 18°C to 25°C, respectively. In the subsequent week (i.e. 01^st^ Mar to 07^th^ Mar 2020, Week 5) this pandemic spread globally.

One of the pivotal reasons for observing such a spread is the frequent mobility of people that caused a sudden surge in COVID-19 infections and led to a global outbreak.

The geographical distributions of total deaths related to COVID-19 are represented in S3 Fig. As of 25 JAN 2020, a cumulative total of 41 deaths were reported in China with an additional of 218 deaths in the following week (26^th^ JAN to 01^st^ FEB 2020).

After one and a half months from the first official reporting (i.e. during 23^rd^ FEB to 29^th^ FEB 2020), 2,837 deaths were reported in China followed by three deaths in Iran, 21 deaths in Italy and 16 deaths in South Korea. After two and a half months of reporting (during 22^nd^ MAR to 28^th^ MAR 2020), maximum deaths were reported from Italy, followed by Spain and China. As of 03 APR 2020, more than 13,000 deaths were reported in Italy followed by Spain and the USA.

### Association of temperature and BCG vaccination with the incidence of new cases

**Fig 2:**
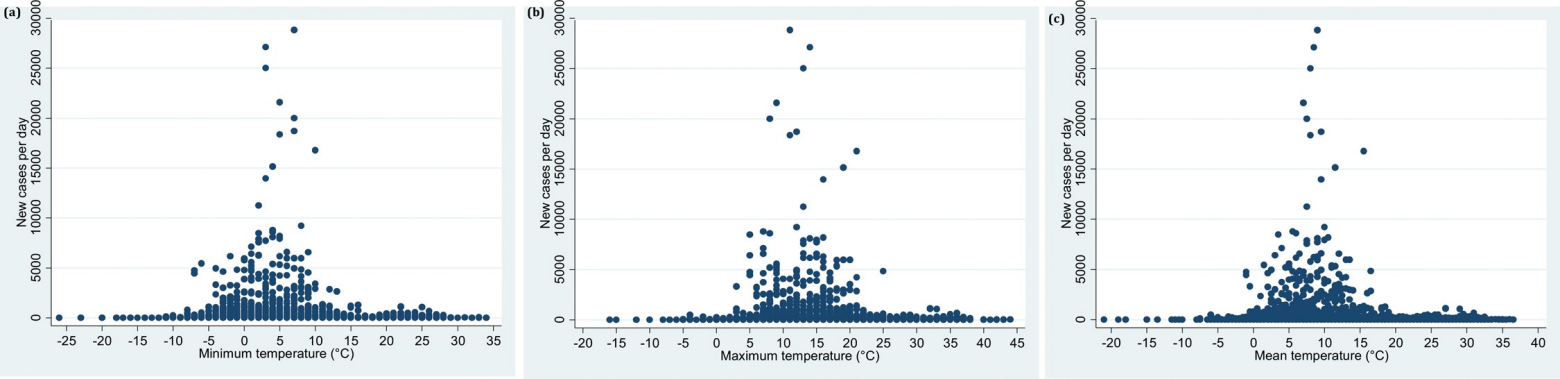
**a:** Distribution of minimum temperature and confirmation of daily new cases from the date of the first case reported till 03^rd^ April, 2020. **b:** Distribution of maximum temperature and confirmation of daily new cases from the date of the first case reported till 03^rd^ April, 2020. **c:** Distribution of mean temperature and confirmation of daily new cases from the date of the first case reported till 03^rd^ April, 2020.

In the present study, bivariate data analysis between the number of new cases and mean temperature depicted that a maximum number of subjects were reported from the regions where the mean temperature ranged between 6°C and 10.5°C till 03^rd^ April 2020 (Table 1 and Fig 2A).

**Table 1:** Minimum and maximum, median and interquartile distribution of new cases in different quartiles.

**Mean temperature**				
	**Median (IQR), Q1, Q3**	**Min**	**Max**	**N**
Quartile 1 (-21º to 5.5º)	11(125), 0, 125	0	8789	798
Quartile 2 (6 º to 10.5 º)	26(237), 1, 238	0	28819	703
Quartile 3 (11 º to 18.5 º)	17.5(95.5), 1, 96.5	0	16797	672
Quartile 4 (19 º to 36.5 º)	5.5(55), 0, 55	0	1138	724
**Total**	14(106), 0 106	0	28819	2897
**Minimum temperature**				
	**Median (IQR), Q1, Q3**	**Min**	**Max**	**N**
Quartile 1 (-26º to 1º)	23(173), 0, 173	0	7123	810
Quartile 2 (2º to 6 º)	16.5(136), 0,136	0	27103	748
Quartile 3 (7 º to 13 º)	15(100), 1,101	0	28819	633
Quartile 4 (14 º to 34 º)	6(47), 0, 47	0	1289	706
**Total**	14(106), 0,106	0	28819	2897
**Maximum temperature**				
	**Median (IQR), Q1, Q3**	**Min**	**Max**	**N**
Quartile 1 (-16º to 9º)	7(105), 0, 105	0	21595	755
Quartile 2 (10º to 16 º)	27.5(219), 1, 220	0	28819	796
Quartile 3 (17 º to 25 º)	20(99), 2, 101	0	16797	657
Quartile 4 (26 º to 44 º)	5(55), 0, 55	0	1138	689
**Total**	14(106), 0, 106	0	28819	2897

**Abbreviations**: IQR: interquartile range, Q1, First quartile, Q3, third quartile, N: Total number of observations

**Mean temperature and BCG vaccination (Model 1).** Our multivariable analysis suggested 1.18 times higher incidence rate ratio in countries, but not statistically significant, where the mean of mean temperature fell in the second quartile (6°C to 10.5°C) compared with the temperature that lied in the first quartile [mean temperature (-21°C to 5.5°C)] (IRR 1.18, 95% CI 0.92 to 1.52). For the countries where the mean temperature fell in the range of 19°C to 36.5°C, the incidence rate was observed to be statistically non-significant with 1.12 times higher incidence as compared with the countries falling in the first quartile (IRR 1.12, 95% CI 0.67 to 1.88) (Table 2, Model 1, Fig 2). The countries that had BCG vaccination coverage had a statistically significant less daily incident cases as compared with countries without BCG coverage in the national immunization program in the adjusted multilevel negative binomial regression analysis (IRR 0.46, 95% CI 0.25 to 0.87).

**Table 2:** Multilevel negative binomial regression analysis to determine the association between daily incident cases and temperature.

**Mean Temperature (model 1)**					
	**IRR (95%CI) Unadjusted**	IRR (95%CI) Adjusted*	**Coefficient**	**P value**	**Z**
**Quartile 1 (-21º to 5.5º)**	**Reference **	**Reference**			
**Quartile 2 (6 º to 10.5 º)**	1.16 (0.91 to 1.48)	1.18 (0.92 to 1.52)	0.17	0.188	1.32
**Quartile 3 (11 º to 18.5 º)**	1.01 (0.73 to 1.41)	1.11 (0.78 to 1.59)	0.10	0.550	0.60
**Quartile 4 (19 º to 36.5 º)**	0.82 (0.50 to 1.36)	1.12 (0.67 to 1.88)	0.11	0.663	0.44
**BCG vaccine**	0.35 (0.18 to 0.66)	0.46 (0.25 to 0.87)	-0.76	0.016	-2.41
**Minimum Temperature (model 2)**					
	**IRR (95%CI) Unadjusted**	IRR (95%CI) Adjusted*	**Coefficient**	**P value**	**Z**
**Quartile 1 (-26º to 1º)**	**Reference**	**Reference**			
**Quartile 2 (2º to 6 º)**	0.71 (0.56 to 0.90)	0.72 (0.57 to 0.93)	-0.32	0.011	-2.56
**Quartile 3 (7 º to 13 º)**	0.69 (0.49 to 0.98)	0.85 (0.59 to 1.24)	-0.15	0.405	-0.83
**Quartile 4 (14 º to 34 º)**	0.55 (0.34 to 0.91)	0.77 (0.46 to 1.29)	-0.25	0.329	-0.98
**BCG vaccine**	0.35 (0.18 to 0.66)	0.50 (0.27 to 0.94)	-0.69	0.032	-2.15
**Maximum Temperature (model 3)**					
	**IRR (95%CI) Unadjusted**	IRR (95%CI) Adjusted*	**Coefficient**	**P value**	**Z**
**Quartile 1 (-16º to 9º)**	**Reference**	**Reference**			
**Quartile 2 (10º to 16 º)**	1.25 (0.99 to 1.57)	1.29 (1.02 to 1.63)	0.25	0.034	2.12
**Quartile 3 (17 º to 25 º)**	1.25 (0.93 to 1.68)	1.37 (0.99 to 1.87)	0.31	0.051	1.95
**Quartile 4 (26 º to 44 º)**	0.89 (0.54 to 1.46)	1.22 (0.73 to 2.04)	0.19	0.448	0.76
**BCG vaccine**	0.35 (0.18 to 0.66)	0.45 (0.24 to 0.84)	-0.79	0.013	-2.50

**Abbreviations:** IRR- Incident Rate Ratio (Ratio between two incidence densities (rate in the exposed divided by rate in unexposed); CI- Confidence Interval; BCG- Bacillus Calmette–Guérin

*Adjusted or total number of tests done for COVID-19 in each country

#### Minimum temperature and BCG vaccination (Model 2)

The countries where the mean of minimum temperature lied between the 2°C to 6°C had significantly lower incidence rate ratio (0.72, 95% CI 0.57 to 0.93) as compared with countries that fell in the first quartile (-26°C to 1°C) which had the maximum number of cases. Countries that fell in the fourth quartile (highest minimum temperature 14°C to 34°C) had a non-significant association with the incident rate as compared with countries that fell in the first quartile (IRR 0.77, 95% 0.46 to 1.29) (Table 2, Model 2, Fig 2). The countries that had BCG vaccination coverage had a statistically significant less daily incident cases as compared with countries without BCG coverage in the national immunization program in the adjusted multilevel negative binomial regression analysis (IRR 0.50, 95% CI 0.27 to 0.94).

#### Maximum temperature and BCG vaccination (Model 3)

Countries with maximum temperature between 10°C to 16°C had a maximum number of cases and were found to have 1.29 times higher incident rate ratio as compared with the countries that fell in the first quartile (IRR 1.29, 95% CI 1.02 to 1.63). The countries that fell in the highest maximum temperature range (26ºC to 44ºC) had statistically non-significant lower incident rate ratio as compared with the countries that fell in the first quartile (IRR 1.22, 95% CI, 0.73 to 2.04) (Table 2, Model 3, and Fig 2). The countries that had BCG vaccination coverage had a statistically significant less daily incident cases as compared with countries without BCG coverage in the national immunization program in the adjusted multilevel negative binomial regression analysis (IRR 0.45, 95% CI 0.24 to 0.84).

### Association of temperature and BCG vaccination with mortality count per day

#### Mean temperature and BCG vaccination (Model 4)

Considering the reference of first quartile, the multilevel negative binomial regression analysis suggested 1.15 times higher incidence rate ratio of mortality in countries falling in the second quartile (mean temperature 6° to 10.5°) compared with the temperature that lied in the first quartile (mean temp -21° to 5.5°), but the association was not statistically significant. For the countries where the mean temperature fell in the range of 19°C to 36.5°C, the incidence rate ratio of mortality count was also not found to be statistically significant as compared with countries falling in the first quartile (IRR 1.99, 95% CI 0.61 to 2.34) (Table 3, Model 4). The findings suggest that high temperature in the countries may not be a probable factor for increased mortality count associated with COVID-19. The countries that had BCG vaccination coverage had a statistically borderline significant less daily mortality count as compared with countries without BCG coverage in the national immunization program (IRR 0.43, 95% CI 0.19 to 1.00).

**Table 3:** Multilevel negative binomial regression analysis to determine the association between daily mortality counts and temperature.

**Mean Temperature (model 4)**					
Temperature quartile	**IRR (95%CI) Unadjusted**	IRR (95%CI) Adjusted*	**Coefficient**	**P value**	**Z**
**Quartile 1 (-21º to 5.5º)**	**Reference**	**Reference**			
**Quartile 2 (6 º to 10.5 º)**	1.18 (0.87 to 1.59)	1.15 (0.84 to 1.58)	0.14	0.373	0.89
**Quartile 3 (11 º to 18.5 º)**	0.97 (0.65 to 1.45)	1.12 (0.71 to 1.77)	0.11	0.612	0.51
**Quartile 4 (19 º to 36.5 º)**	0.87 (0.47 to 1.62)	1.99 (0.61 to 2.34)	0.18	0.594	0.53
**BCG vaccine**	0.37 (0.17 to 0.79)	0.43 (0.19 to 1.00)	-0.83	0.050	-1.96
**Minimum Temperature (model 5)**					
Temperature quartile	**IRR (95%CI) Unadjusted**	IRR (95%CI) Adjusted*	**Coefficient**	**P value**	**Z**
**Quartile 1 (-26º to 1º)**	**Reference**	**Reference**			
**Quartile 2 (2º to 6 º)**	0.62 (0.46 to 0.84)	0.62 (0.45 to 0.85)	-0.47	0.003	-2.95
**Quartile 3 (7 º to 13 º)**	0.66 (0.43 to 1.00)	0.75 (0.47 to 1.21)	-0.27	0.248	-1.15
**Quartile 4 (14 º to 34 º)**	0.75 (0.40 to 1.37)	0.96 (0.50 to 1.86)	-0.035	0.916	-0.11
**BCG vaccine**	0.37 (0.17 to 0.79)	0.47 (0.20 to 1.09)	-0.76	0.078	-1.77
**Maximum Temperature (model 6)**					
Temperature quartile	**IRR (95%CI) Unadjusted**	IRR (95%CI) Adjusted*	**Coefficient**	**P value**	**Z**
**Quartile 1 (-16º to 9º)**	**Reference**	**Reference**			
**Quartile 2 (10º to 16 º)**	1.34(1.01 to 1.76)	1.37 (1.02 to 1.83)	0.31	0.038	2.08
**Quartile 3 (17 º to 25 º)**	1.24(0.86 to 1.79)	1.40 (0.94 to 2.10)	0.34	0.095	1.67
**Quartile 4 (26 º to 44 º)**	1.11 (0.60 to 2.06)	1.46 (0.75 to 2.84)	0.38	0.259	1.13
**BCG vaccine**	0.37 (0.17 to 0.79)	0.42 (0.18 to 0.95)	-0.87	0.037	-2.09

**Abbreviations:** IRR- Incident Rate Ratio (Ratio between two incidence densities (rate in the exposed divided by rate in unexposed), CI- Confidence Interval; BCG- Bacillus Calmette–Guérin

*Adjusted or total number of tests done for COVID-19 in each country

#### Minimum temperature and BCG vaccination (Model 5)

The countries that had the highest mean of minimum temperature (14°C to 34°C) did not statistically differ in the incident rate ratio of mortality as compared with countries that fell in the first quartile (IRR 0.96, 95% CI 0.50 to 1.86) (Table 3, Model 5). The countries that had BCG vaccination coverage had a statistically non-significant less daily mortality count as compared with countries without BCG coverage in the national immunization program (IRR 0.47, 95% CI 0.20 to 1.09).

#### Maximum temperature and BCG vaccination (Model 6)

**Fig 3:**
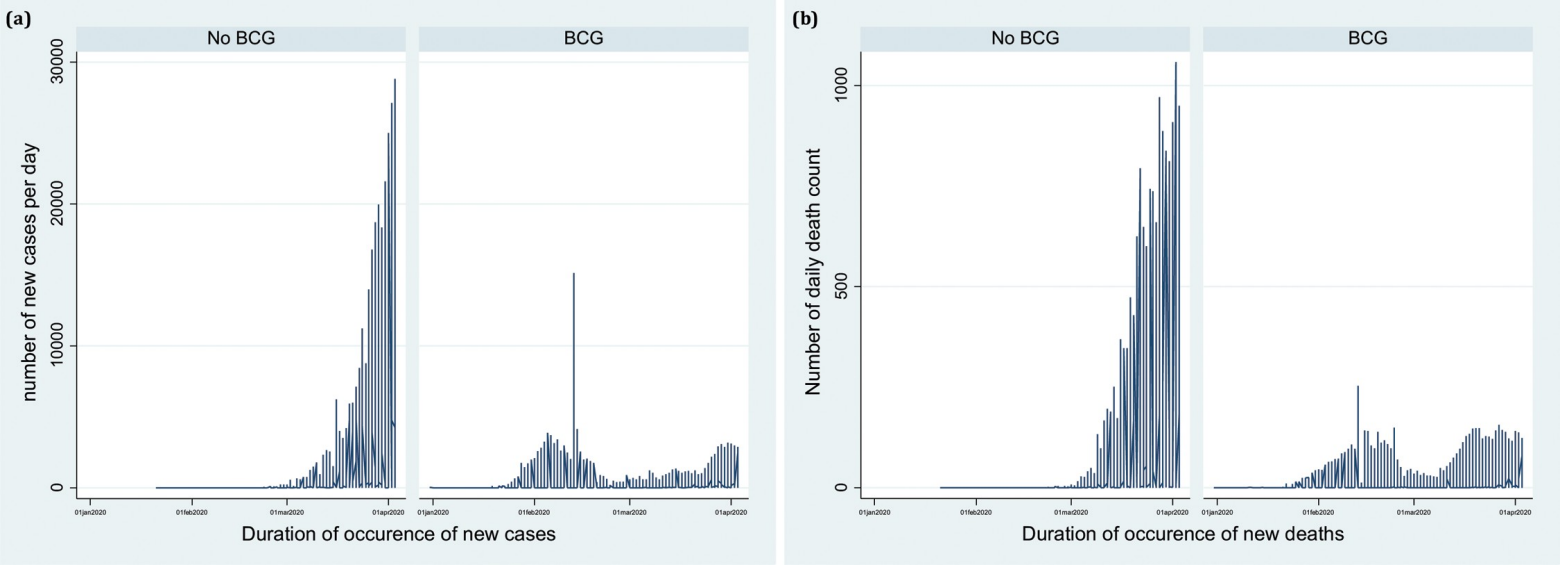
**a:** Distribution of daily reported cases due to COVID-19 with or without the neonatal BCG vaccination coverage. **b:** Distribution of daily reported mortality due to COVID-19 with or without the neonatal BCG vaccination coverage.

The countries that fell in the second quartile (maximum temperature in a range of 10°C to 16°C) had 1.37 times higher mortality compared with countries falling in the first quartile. The countries that had the highest maximum temperature (26°C to 44°C) did not statistically differ in mortality count as compared with countries falling in the first quartile (IRR 1.46, 95% CI 0.75 to 2.84) (Table 3, Model 6). The countries that had BCG vaccination coverage had a statistically significant less mortality count as compared with countries without BCG coverage in the national immunization program in the adjusted multilevel negative binomial regression analysis (IRR 0.42, 95% CI 0.18 to 0.95) (Table 3, Model 6). These findings suggest BCG vaccination coverage could be a potential factor for less spread of COVID-19 infection in the respective countries. A time-series graph also supported the notion that a smaller number of cases and less mortality were present in the countries where BCG vaccination is currently under the immunization program (Fig 3A and 3B).

## Discussion

The present study was conducted to investigate the relationship of human-to-human transmission of COVID-19 infection with temperature and neonatal BCG vaccination in the immunization program across the countries worldwide. Our analysis showed a non-linear pattern of transmission of COVID-19 with temperature, indicating that the most feasible temperature for the transmission is the lower range of temperature. The countries where maximum numbers of new cases were observed had the mean temperature in the range of 6°C to 10.5°C. The number of cases decreased beyond the 18.5°C temperature threshold. However, we did not observe the statistically significant association of mean temperature and spread of the COVID-19. The findings of the current study observed discordant pattern, as observed in the previously published studies [22, 30, 31]. Our study consisted of updated data till 03 April, 2020 and by that time reliable number of cases were reported to draw any inference. However, our study further strengthens this notion by investigating the association of temperature with the spread of COVID-19 virus using robust statistical modelling. We have used multilevel negative binomial regression to determine the probability associated with temperature and COVID-19 virus transmission. We adjusted the effect of the number of COVID-19 tests performed in respective countries to exclude their confounding effect. The more we test, the more likely we are to capture the COVID-19 positive cases; in other words, a greater number of cases were observed in countries where maximum numbers of tests were done. The number of COVID-19 virus tests done to detect the COVID-19 in a population has been covered in the reports published by several private and public information sources, and various online databases of several government agencies and also indicated by the World Health Organisation [32, 33]. The findings of the present study did not support the hypothesis that higher temperature might have a role in less spread of COVID-19 after adjusting the confounding effect of the number of tests done in respective countries in the multilevel negative binomial regression model. A large scale of transmission of COVID-19 has been noted across different countries worldwide, and most of these countries had a diverse range of temperature, including low and high temperature. The temperature has been observed to be the most important established factor for the transmission and survival of SARS-CoV, MERS-CoV and influenza virus [5, 19]. Cold temperature was found to be the optimal temperature for prolonged half-life, stabilization of droplet and enhanced propagation in nasal mucosa for other respiratory viruses [34]. A recent study published by Yao Y *et al*., in 2020 also observed no association of COVID-19 transmission with temperature or UV radiation in Chinese cities [35]. However, the generalizability of their findings is questionable since that study had several caveats. The data were presented for only a single country, i.e. China, where the outbreak initiated. Further, the study included only a limited number of cities with temperature more than 20 degrees. Therefore, the possibility of missing the true effect cannot be denied, and thus, their findings must be interpreted with caution. Our study includes the temperature data of cities from 67 distinct countries and did not support that high average temperature may be associated with less transmissibility of COVID-19.

In the upcoming days, the temperature has risen drastically in most of the countries, especially in the tropical regions and larger numbers of cases have been reported from the tropical countries also. The human coronaviruses which typically causes common cold have been observed to exhibit strong winter seasonality between December and April and are undetectable in summer months. A study observed that HCoV-OC43 (a beta coronavirus of same family COVID-19) had strong winter seasonality [36]. The findings of the present study did not suggest that transmission of COVID-19 will probably diminish in the high-temperature regions with minimum temperature. However, it is difficult to accurately forecast as COVID-19 has appeared for the first time and there is probably no pre-existing immunity. It is expected that World’s mortality rates will continue to increase by 1% in every three weeks [37]. The public health interventions like citywide/nationwide lockdowns would also help in containing the virus and preventing its spread in upcoming months [38]. The predictive models would further allow answering the questions like what are the most potential preventable approaches to limit the transmission of COVID-19; what are the regions in the countries which are most at risk; duration of the transmission so that strict control measures could be adapted to prevent transmission of COVID-19 and also for understanding the potential factors which may limit the transmission of this virus.

Emerging evidence has pointed out the potential of neonatal BCG vaccination as a promising tool in the fight against the COVID-19 and has been reported to offer a broad range of immunity against the respiratory infection. A recent study found that BCG vaccination is associated with less morbidity and mortality due to COVID-19 in countries where the universal and long-standing childhood BCG vaccination policies are implemented as compared with the countries without universal BCG vaccination policies and concluded BCG vaccination as a potential new tool in the fight against COVID-19 [39]. Different countries have different vaccination schedule depending on their health care policies. We divided the reported case data with or without BCG vaccination policies in the respective countries and analysed the data to check for a similar effect observed in the previously reported study. Our findings supported the notion that frequencies of new cases are substantially higher in the countries where no universal BCG vaccination is present. As far as mortality count is concerned, we observed that the countries where the BCG vaccination coverage was embedded in the national immunization program had approximately 58% less mortality count (model 6) as compared with those countries without BCG vaccination coverage. While performing multilevel negative binomial regression analysis, we have even adjusted for the number of tests performed in respective countries, an important potential confounding factor. Although, we cannot exclude the bias in our observations due to other potential confounders like differences in the national demographics, burden of disease and the stage of the pandemic in each country which could not be adjusted in the multivariable models. Furthermore, we adjusted the number of tests done for detection of COVID-19 which may also be influenced by the national demographics, the burden of disease and the stage of pandemic. Our present study findings provide the evidence to conduct well designed randomized controlled trials to validate these findings before its clinical translation and its further implications. World Health Organisation is also looking forward to future evidence from the two ongoing clinical trials addressing this question [40]. Furthermore, the addition of new cases in the upcoming days might provide more power to detect this effect precisely for obtaining any conclusive remark. The Government initiatives and policies, and the effect of failures to control this pandemic can be modelled and projected accurately provided the trend in the daily cases of COVID-19 and its associated deaths can be updated with an additional environmental temperature and BCG vaccination coverage. Due to the unavailability of information on variables that can explain exact temperature and BCG vaccination coverage, can be explored later on.

### Limitation

The temperature data of most representative capital cities was taken for the respective countries. Individual city temperature data, including their respective number of cases, could not be obtained, thereby limiting to see this effect with cases reported in each city and exposure of temperature during the same time period. Age and sex information for individual case data was not available for many countries which might be an important factor for the transmission of COVID-19 in different age groups. The causality of effect cannot be examined with the available epidemiological data. Human factors like public health intervention, lockdown, travel history, the mutation rate of virus and pathogenesis have not been analysed. Future forecasting of cases has not been done.

## Conclusion

Our exploratory study suggests that probably temperature might not be associated with less transmission of COVID-19. The spread rate was statistically significantly less, with 58% less mortality due to COVID-19 in countries having neonatal BCG vaccination policy. However, the findings of this exploratory study are based on the secondary cross-sectional data, available in the public domain and further well designed randomized controlled trials are needed to detect the cause-and-effect relationship.

## Supporting information

S1 Fig Global transition of infection cases due to COVID-19 reported from Week 1 to Week 11. Weekly geographic distribution worldwide: Week 1 (22 JAN 2020–25 JAN 2020), Week 2 (26 JAN 2020–01 FEB 2020), Week 3 (02 FEB 2020–08 FEB 2020), Week 4 (09 FEB 2020–15 FEB 2020), Week 5 (16 FEB 2020–22 FEB 2020), Week 6 (23 FEB 2020–29 FEB 2020), Week 7 (01 MAR 2020–07 MAR 2020), Week 8 (08 MAR 2020–14 MAR 2020), Week 9 (15 MAR 2020–21 MAR 2020), Week 10 (22 MAR 2020–28 MAR 2020), Week 11 (29 MAR 2020–03 APR 2020).
(DOC)

S2 Fig Global pattern of temperature changes during spreading of COVID-19 from Week 1 to Week 11. Weekly geographic distribution worldwide: Week 1 (22 JAN 2020–25 JAN 2020), Week 2 (26 JAN 2020–01 FEB 2020), Week 3 (02 FEB 2020–08 FEB 2020), Week 4 (09 FEB 2020–15 FEB 2020), Week 5 (16 FEB 2020–22 FEB 2020), Week 6 (23 FEB 2020–29 FEB 2020), Week 7 (01 MAR 2020–07 MAR 2020), Week 8 (08 MAR 2020–14 MAR 2020), Week 9 (15 MAR 2020–21 MAR 2020), Week 10 (22 MAR 2020–28 MAR 2020), Week 11 (29 MAR 2020–03 APR 2020).
(DOC)

S3 Fig Global transition of deaths cases due to COVID-19 reported from Week 1 to Week 11. Weekly geographic distribution worldwide: Week 1 (22 JAN 2020–25 JAN 2020), Week 2 (26 JAN 2020–01 FEB 2020), Week 3 (02 FEB 2020–08 FEB 2020), Week 4 (09 FEB 2020–15 FEB 2020), Week 5 (16 FEB 2020–22 FEB 2020), Week 6 (23 FEB 2020–29 FEB 2020), Week 7 (01 MAR 2020–07 MAR 2020), Week 8 (08 MAR 2020–14 MAR 2020), Week 9 (15 MAR 2020–21 MAR 2020), Week 10 (22 MAR 2020–28 MAR 2020), Week 11 (29 MAR 2020–03 APR 2020).
(DOC)

S1 File (DOCX)
