# Supplementary figures and images for: Global impact of environmental temperature and BCG vaccination coverage on the transmissibility and fatality rate of COVID-19
Source: PLoS One. 2020 Oct 22;15(10):e0240710. doi: 10.1371/journal.pone.0240710 (PMC7580966; doi:10.1371/journal.pone.0240710)

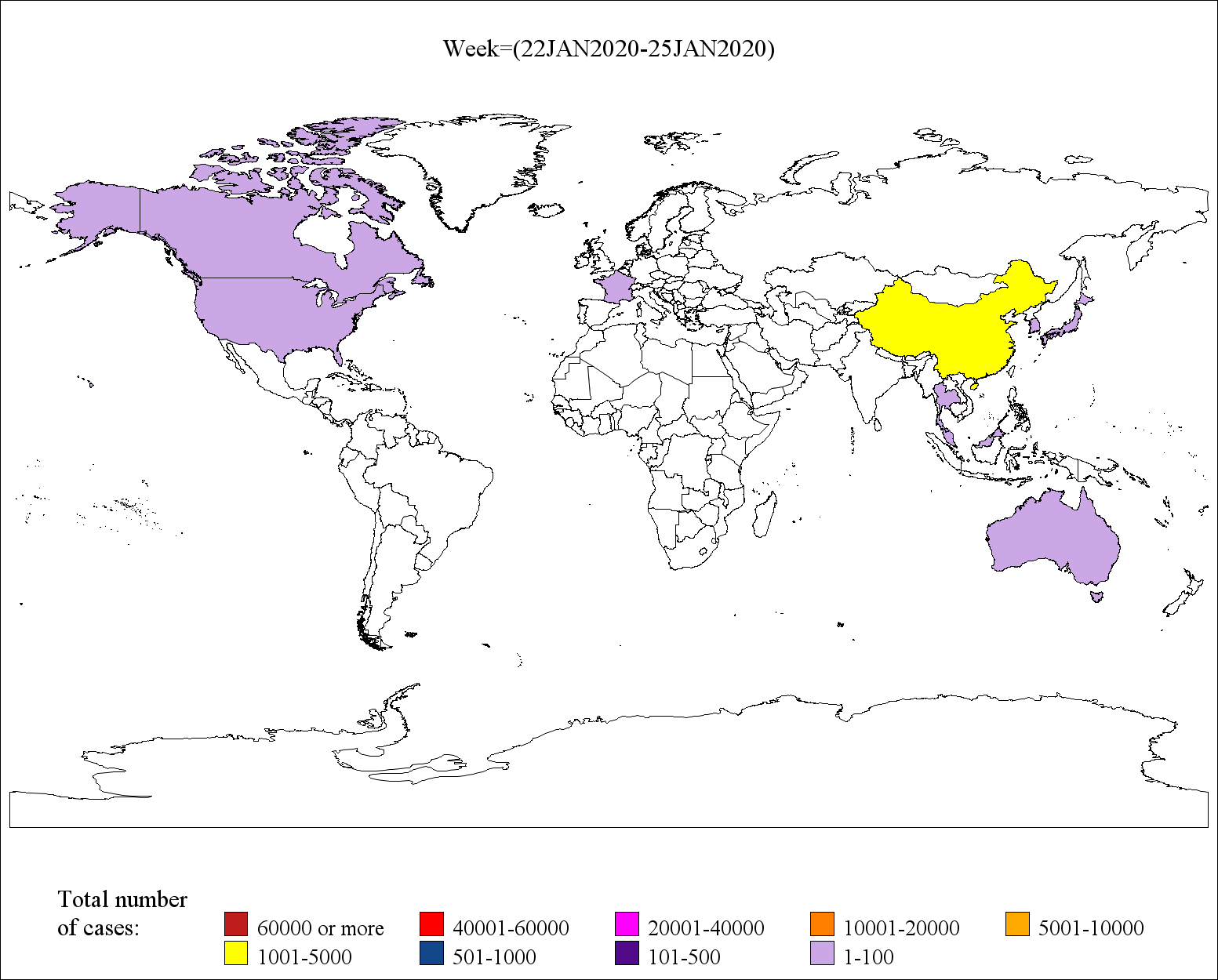


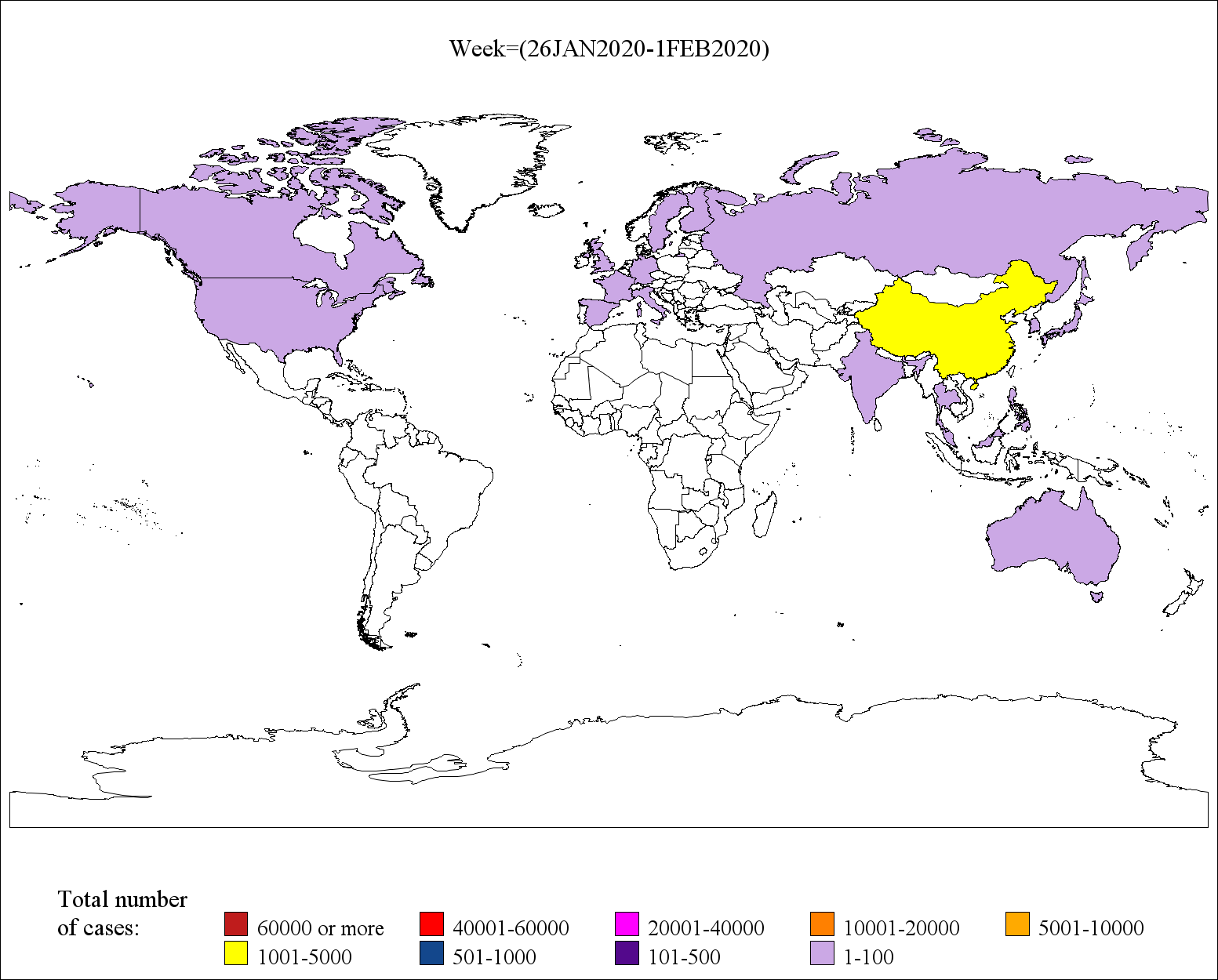


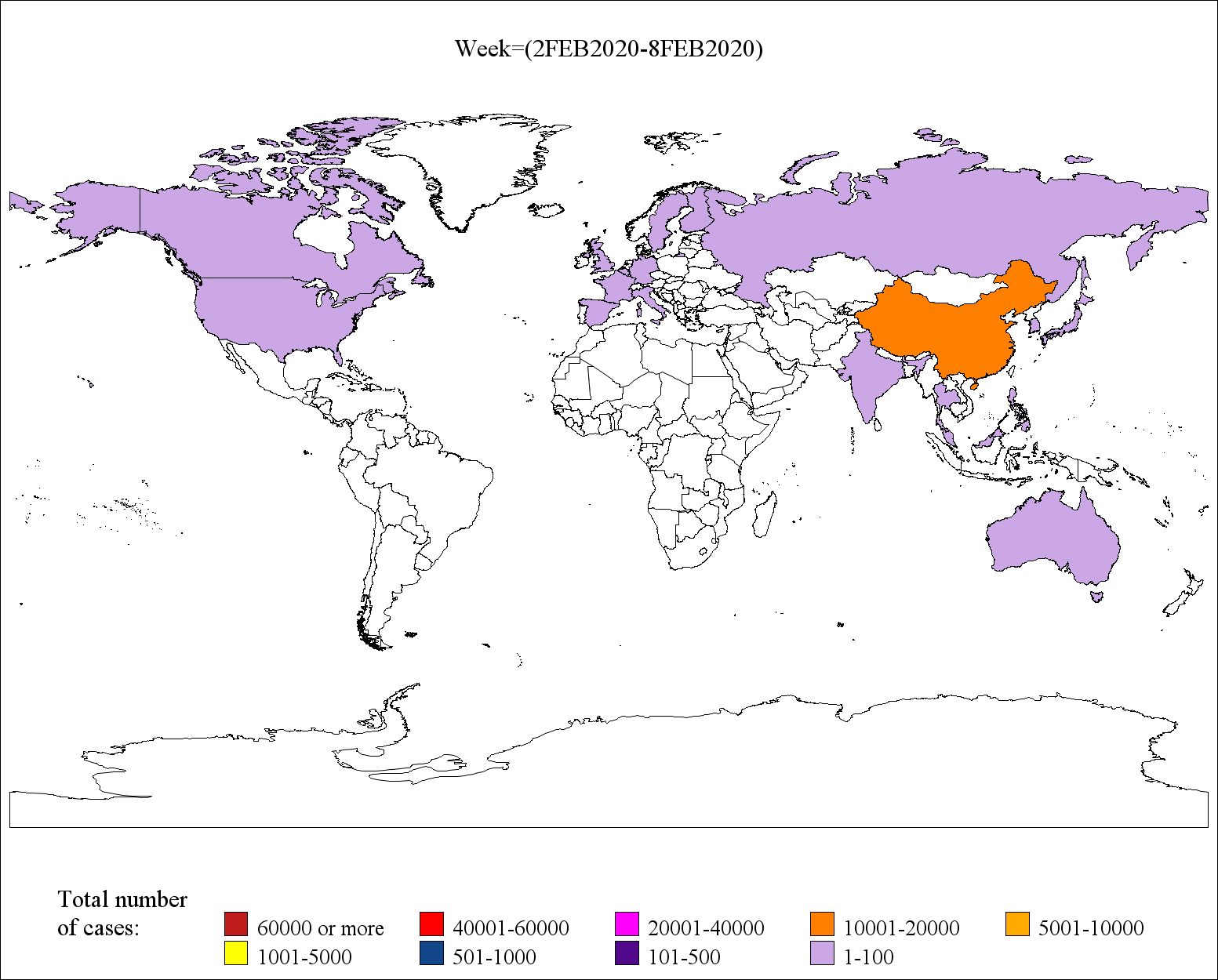


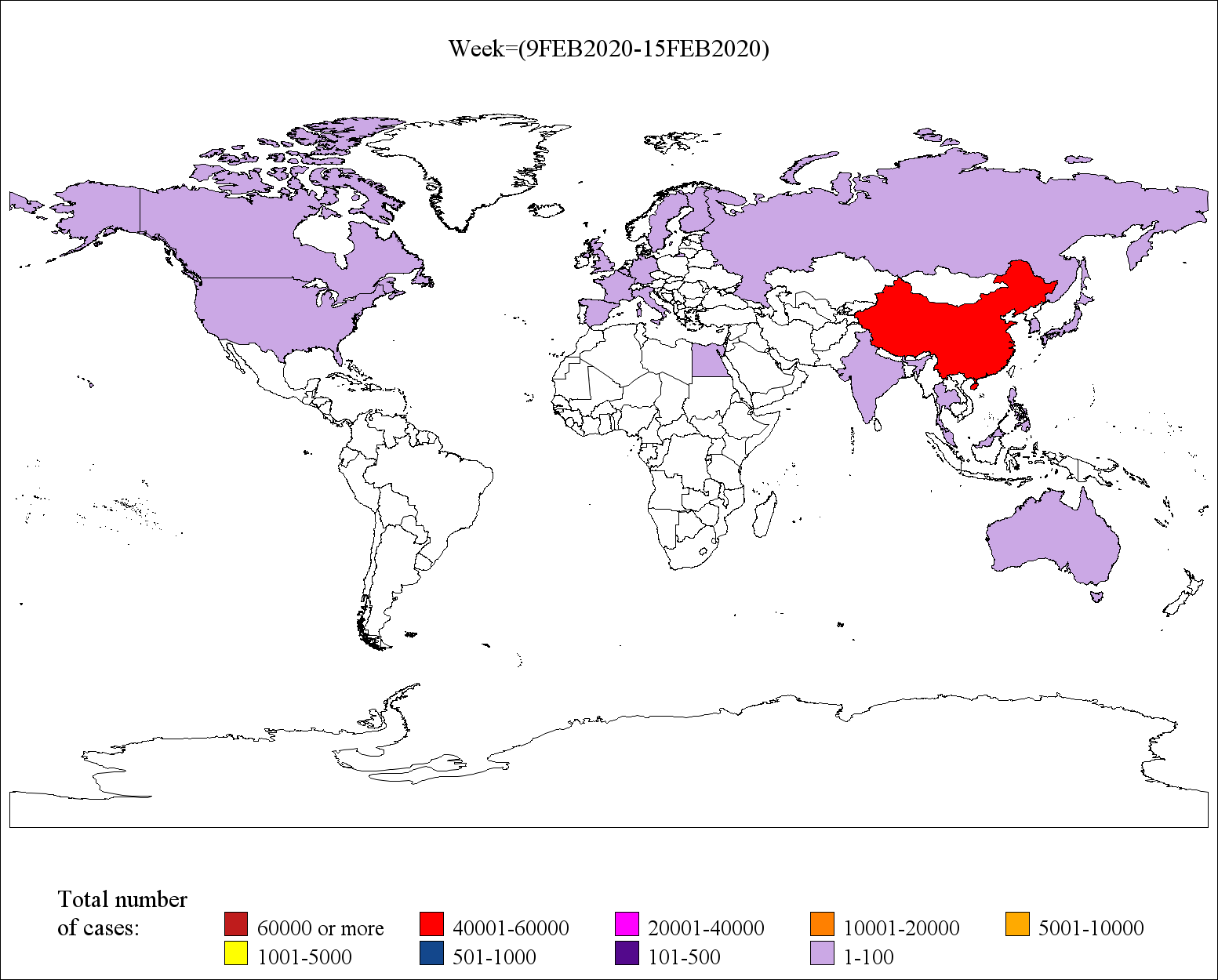


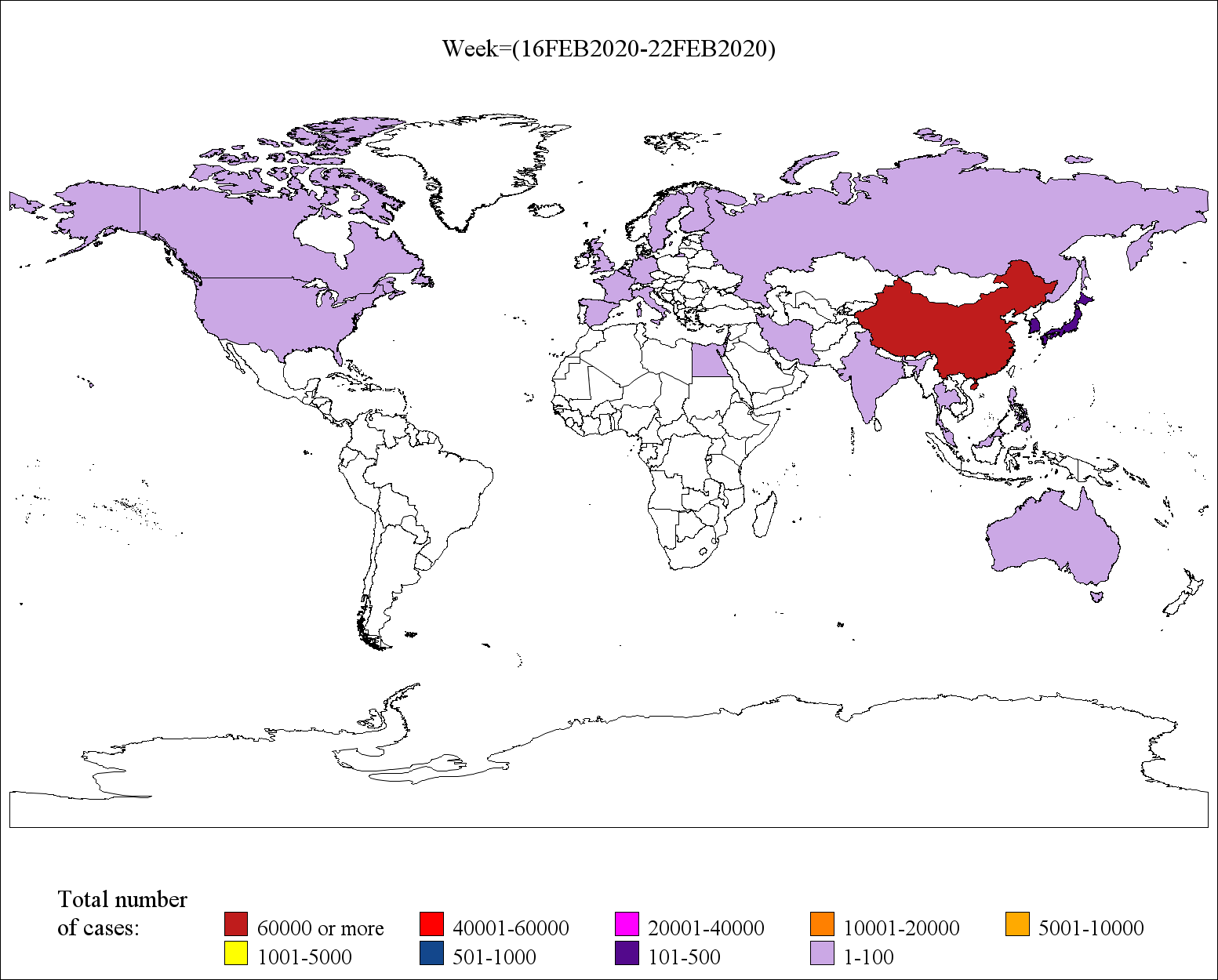


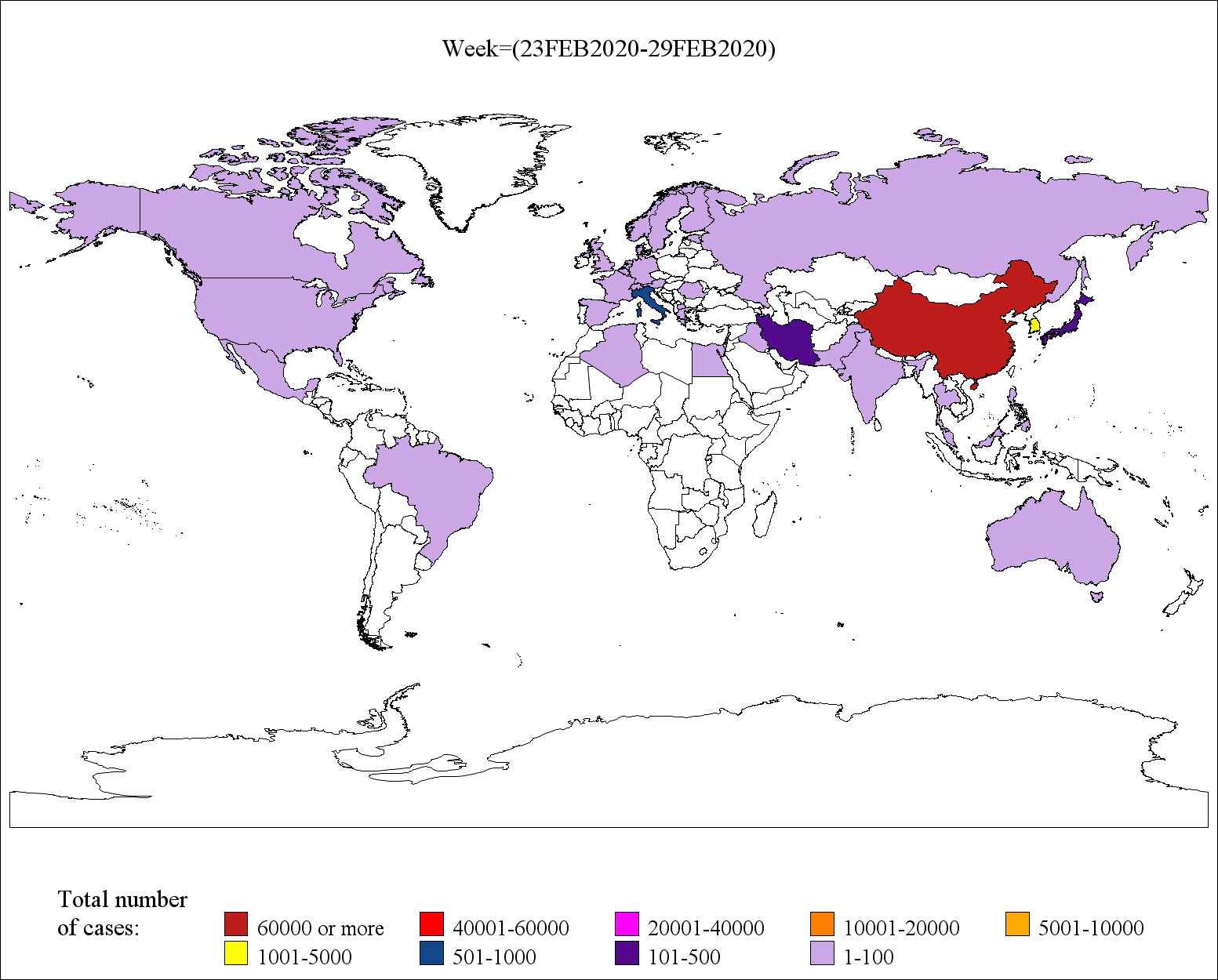


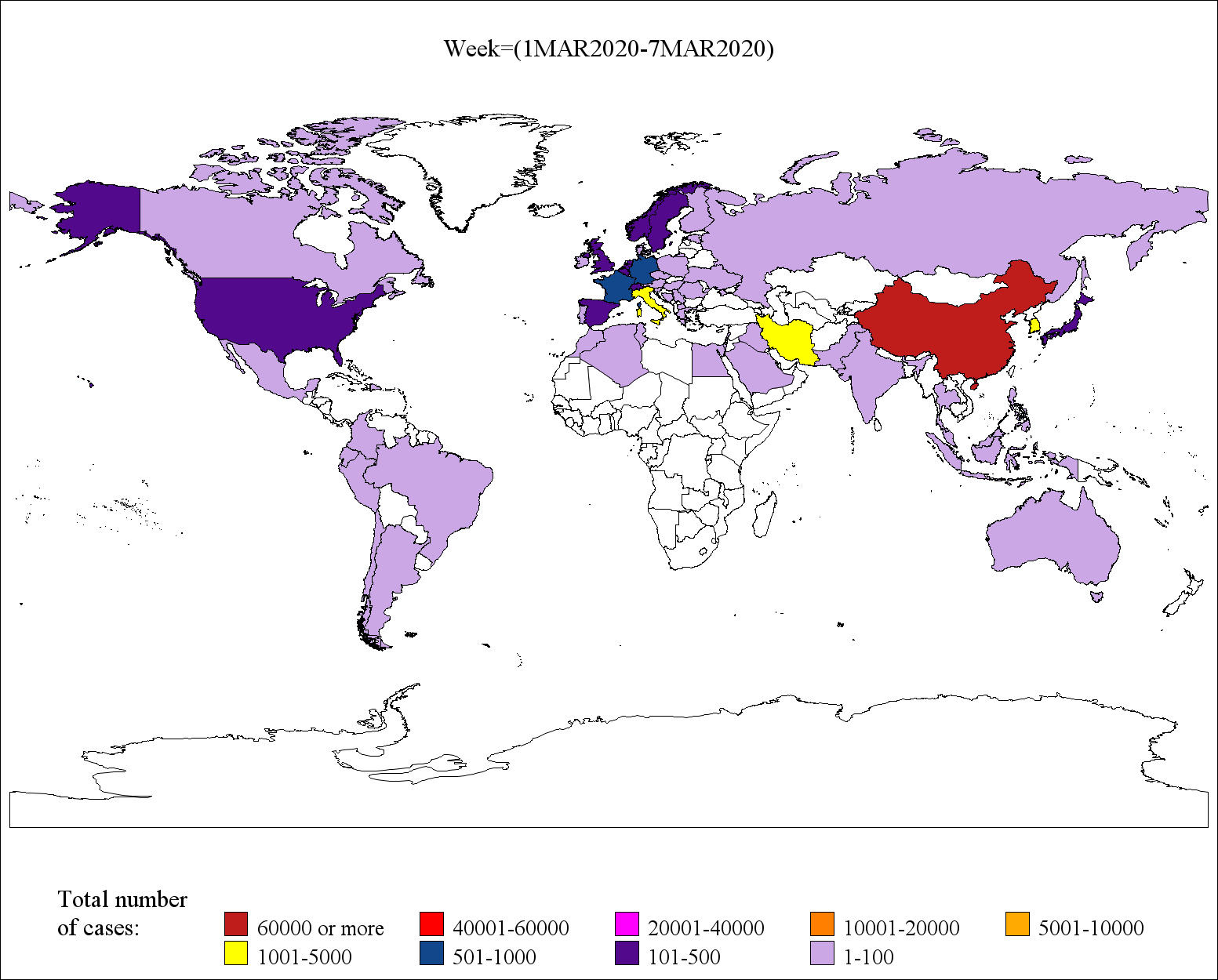


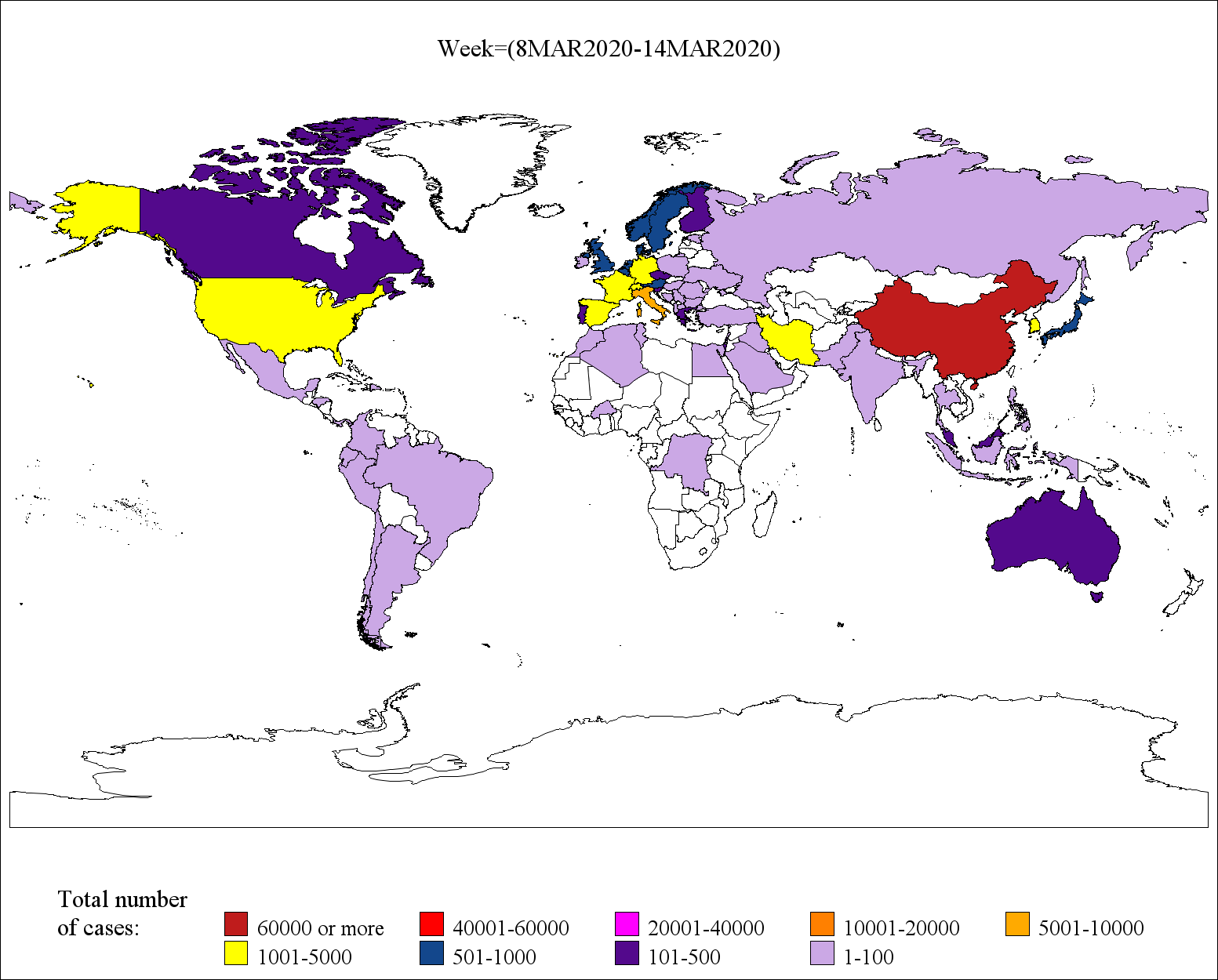


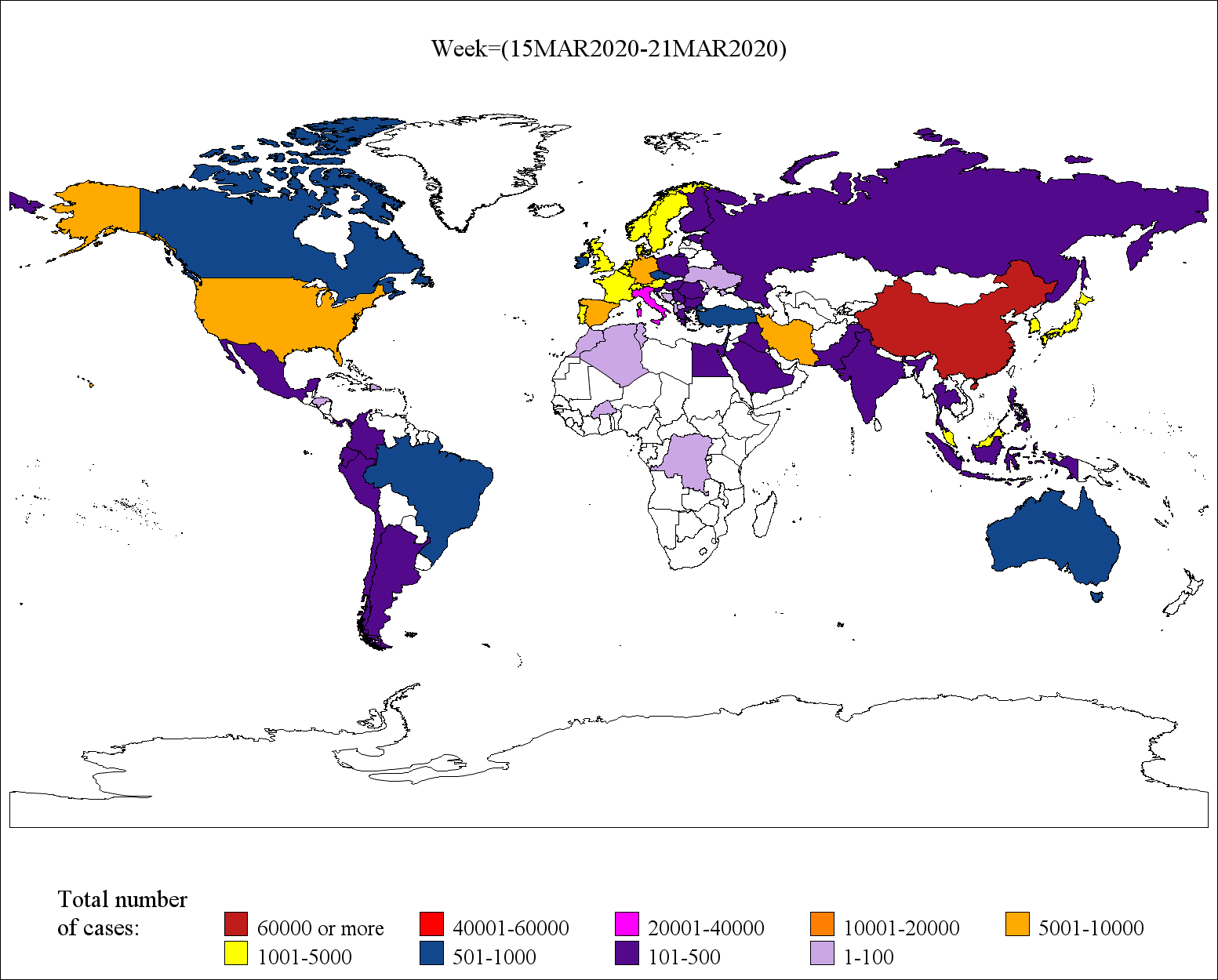


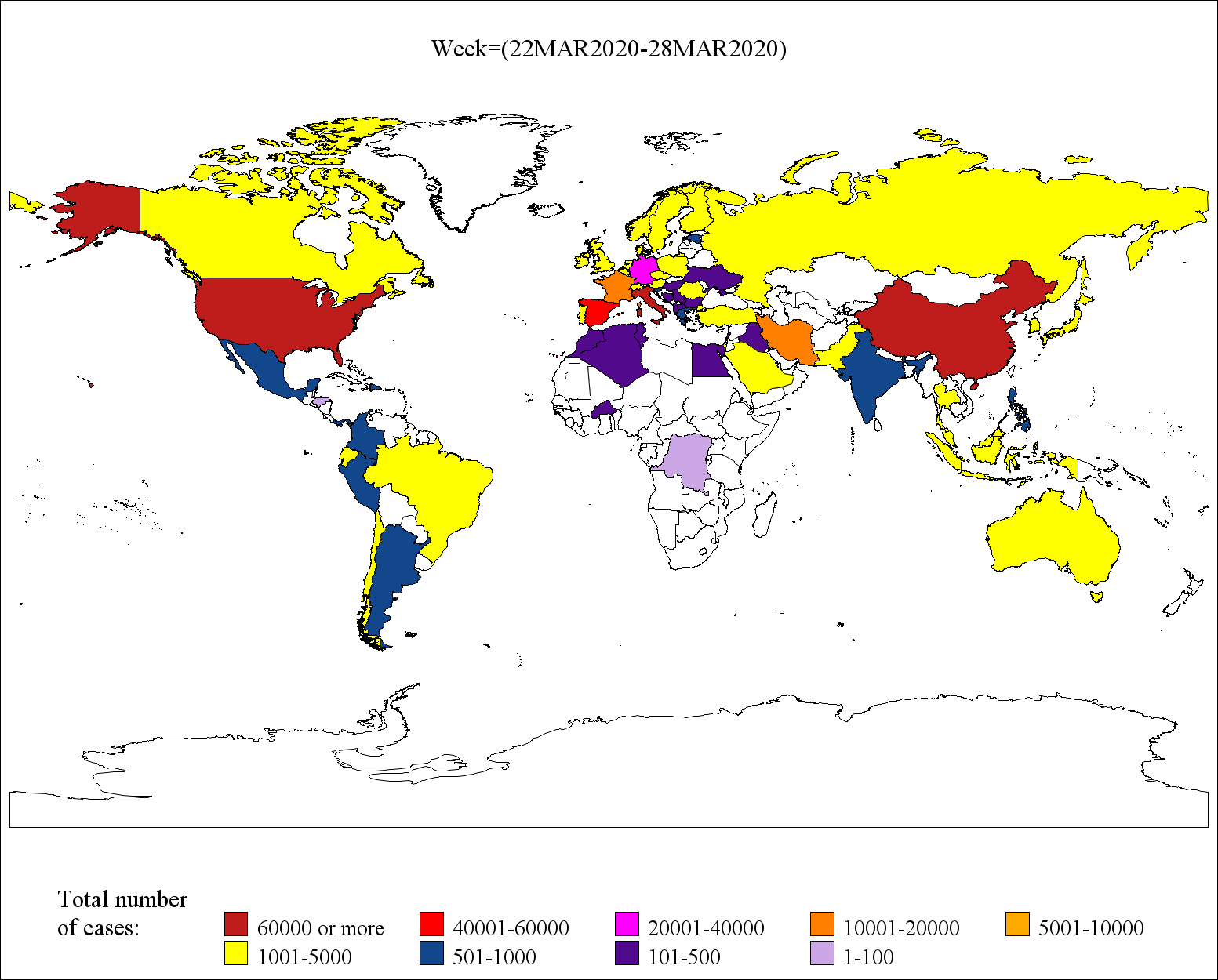


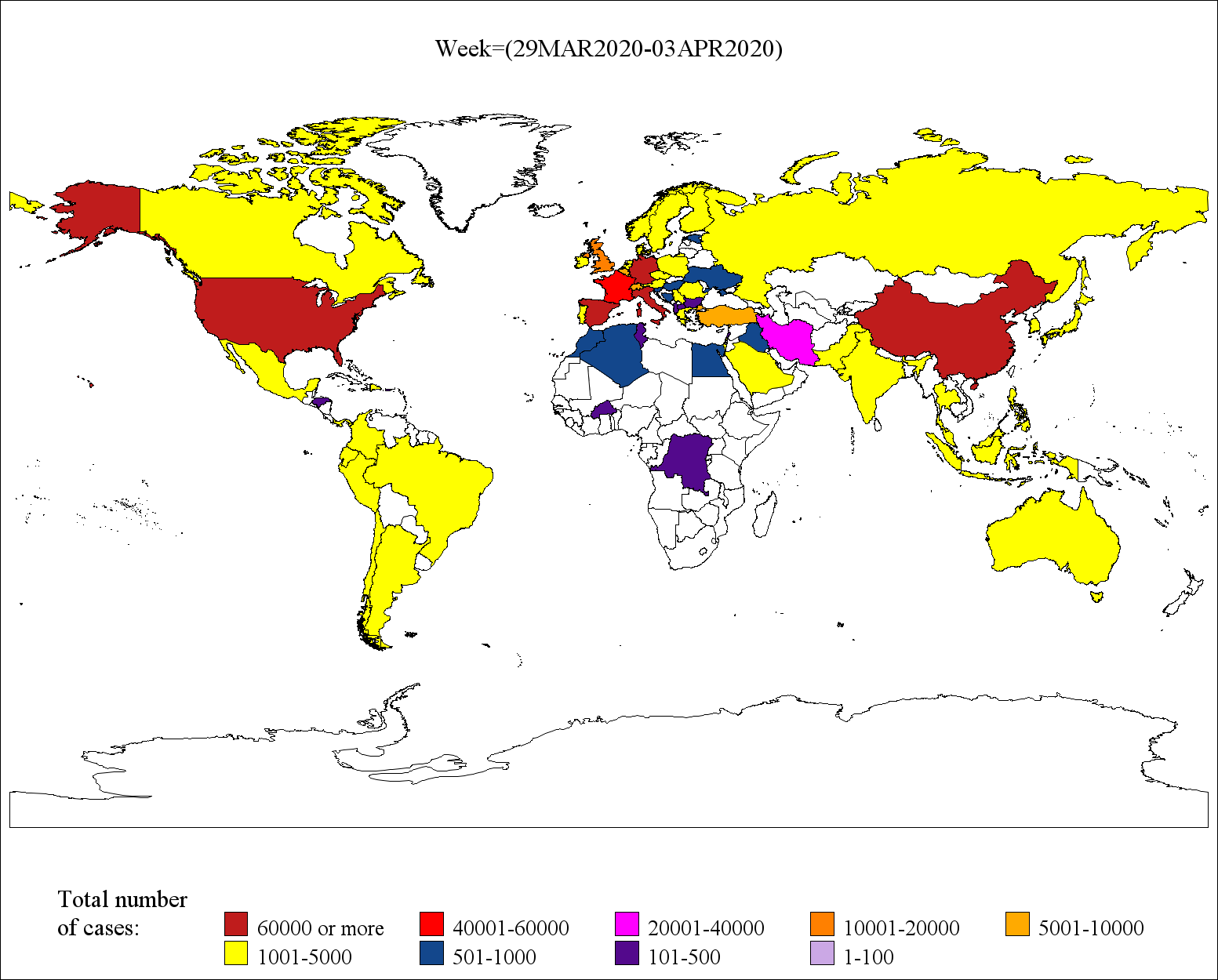

Supplement: S1 Fig — Weekly geographic distribution worldwide: Week 1 (22 JAN 2020–25 JAN 2020), Week 2 (26 JAN 2020–01 FEB 2020), Week 3 (02 FEB 2020–08 FEB 2020), Week 4 (09 FEB 2020–15 FEB 2020), Week 5 (16 FEB 2020–22 FEB 2020), Week 6 (23 FEB 2020–29 FEB 2020), Week 7 (01 MAR 2020–07 MAR 2020), Week 8 (08 MAR 2020–14 MAR 2020), Week 9 (15 MAR 2020–21 MAR 2020), Week 10 (22 MAR 2020–28 MAR 2020), Week 11 (29 MAR 2020–03 APR 2020). (DOC) [file pone.0240710.s001.doc]

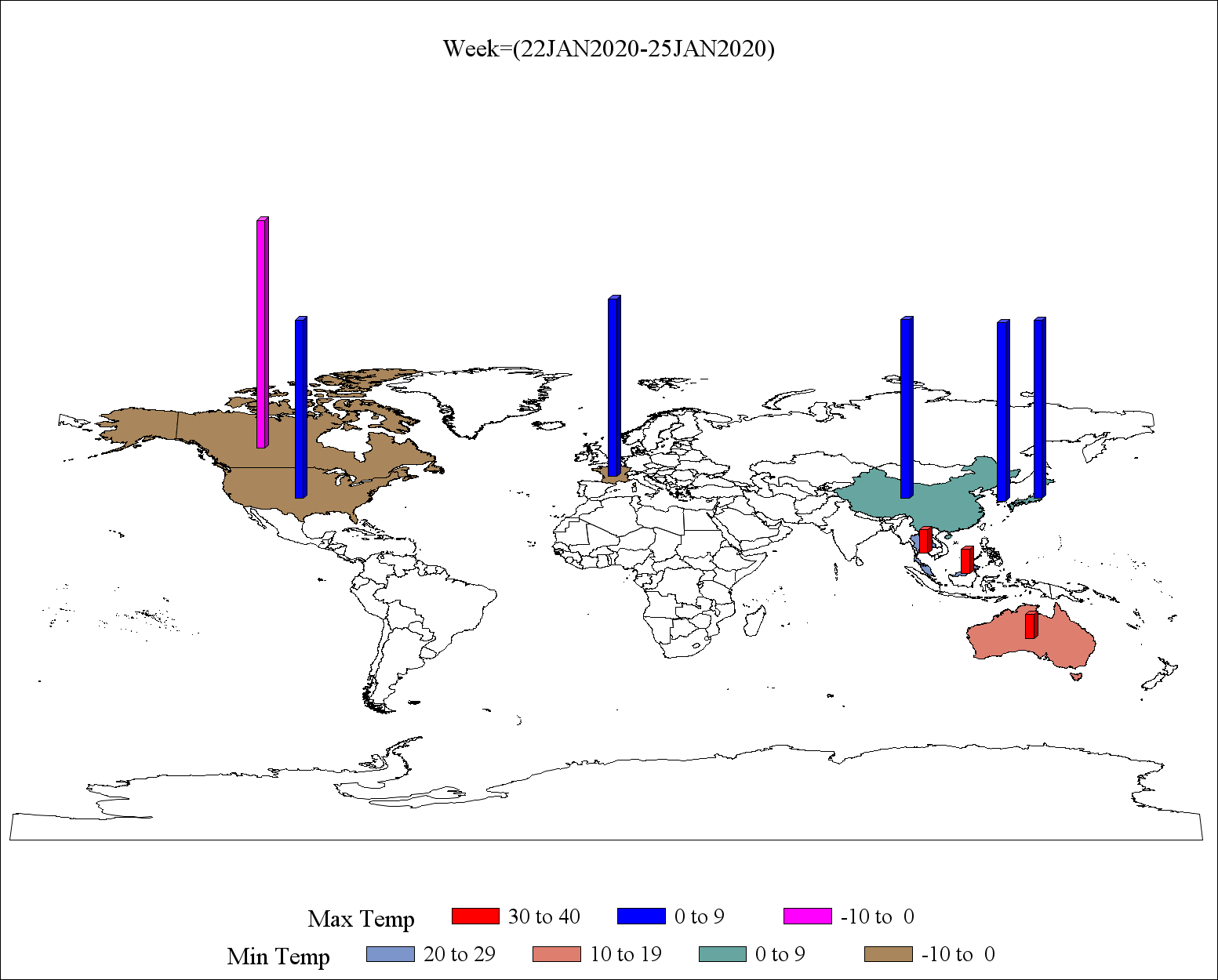


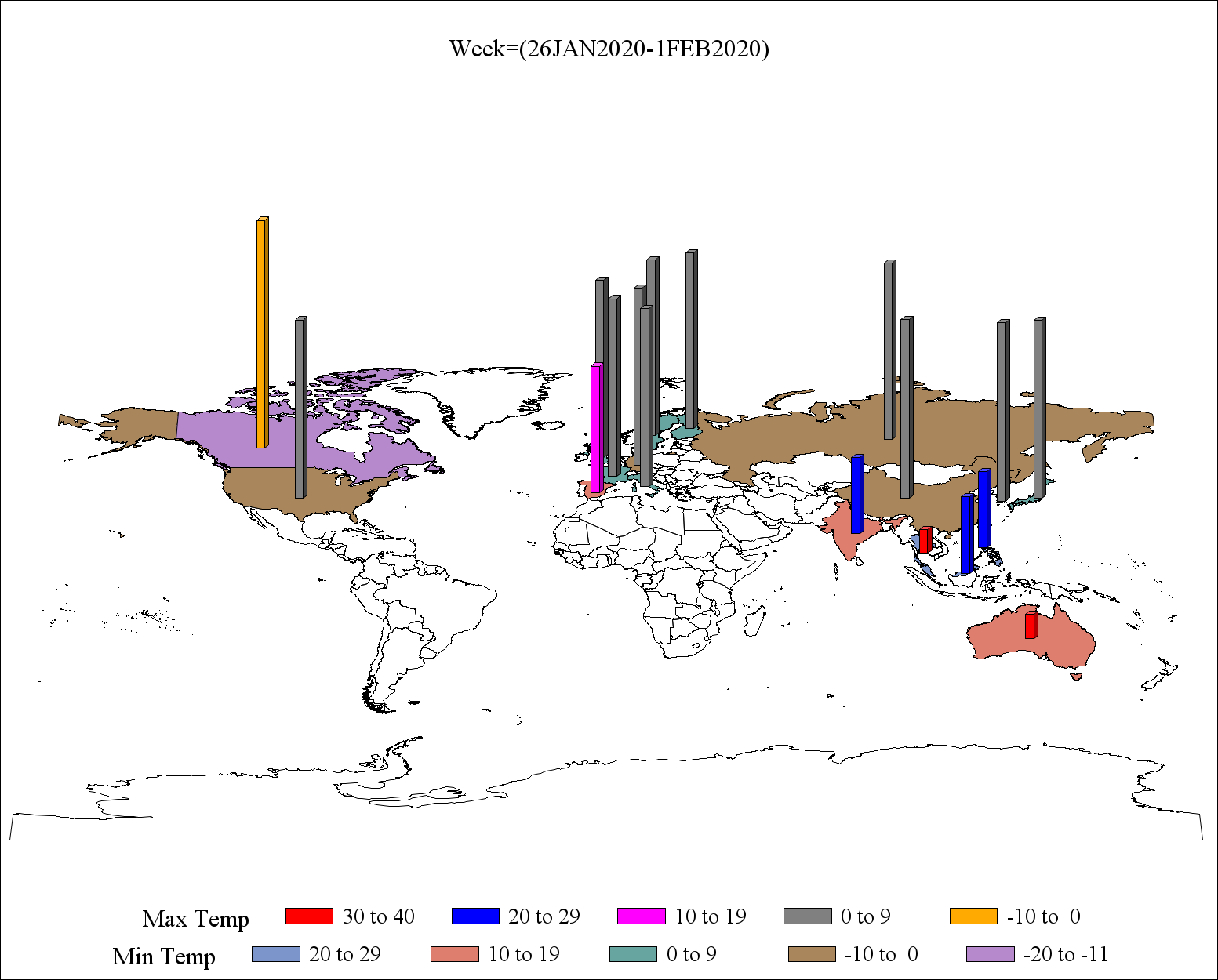


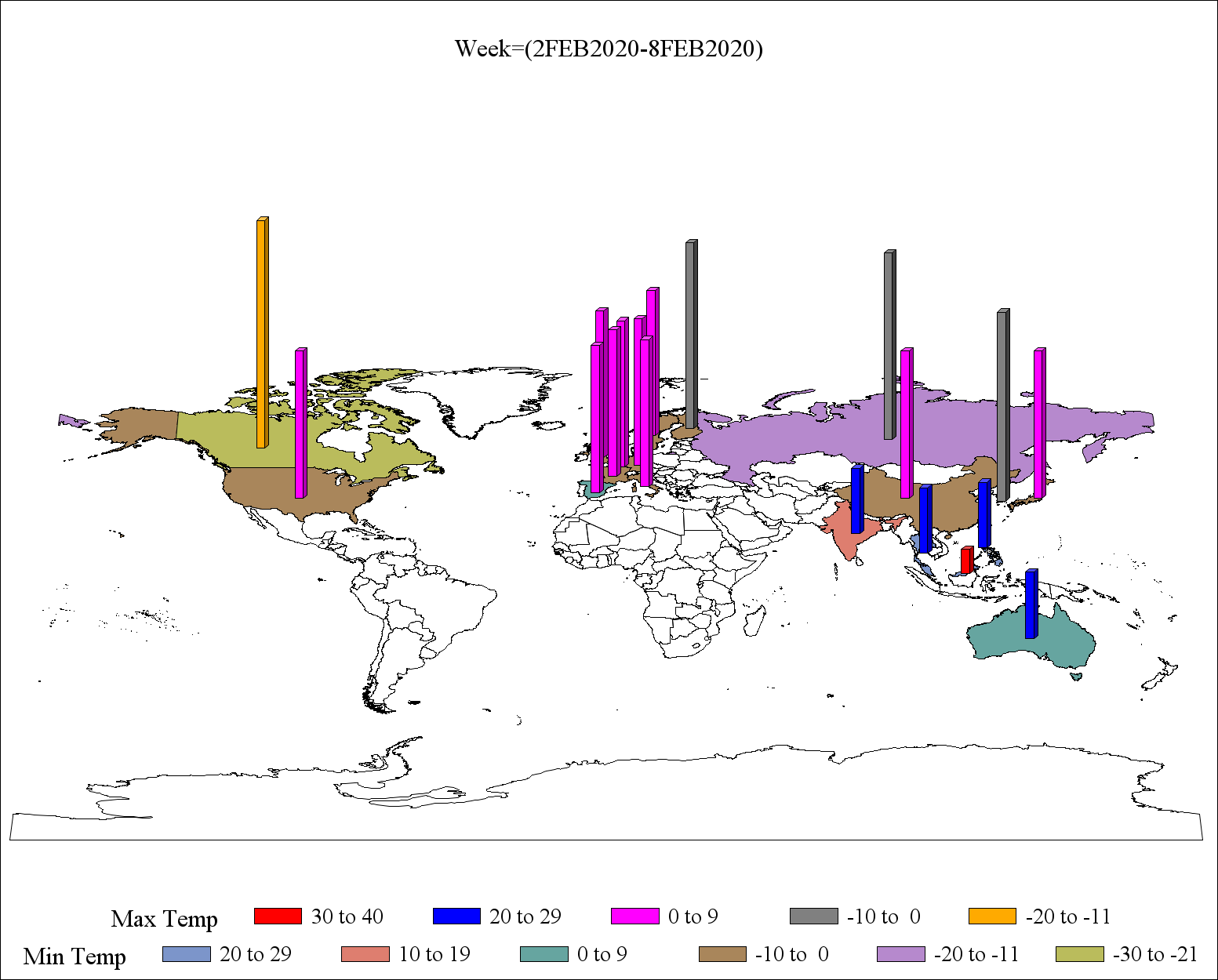


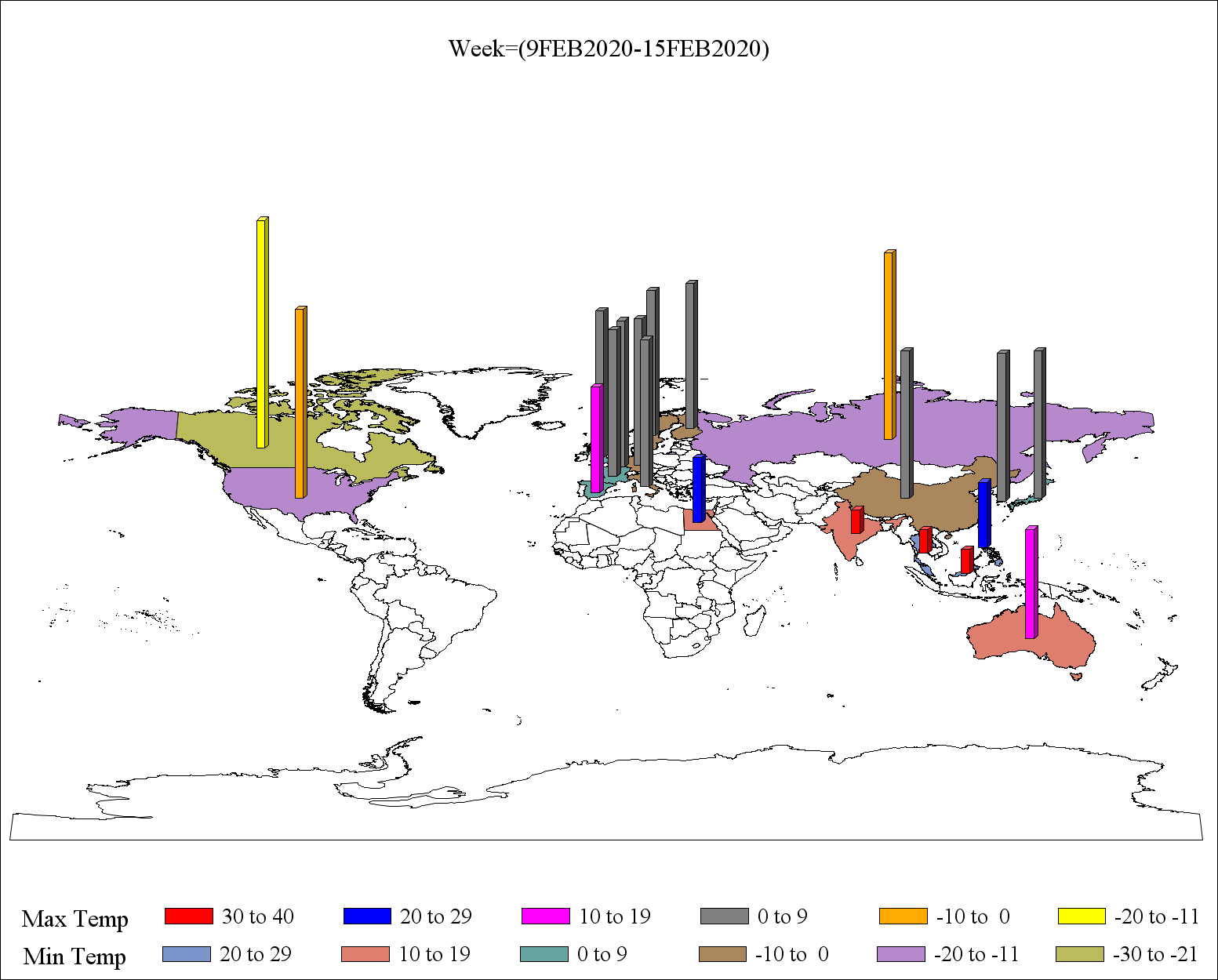


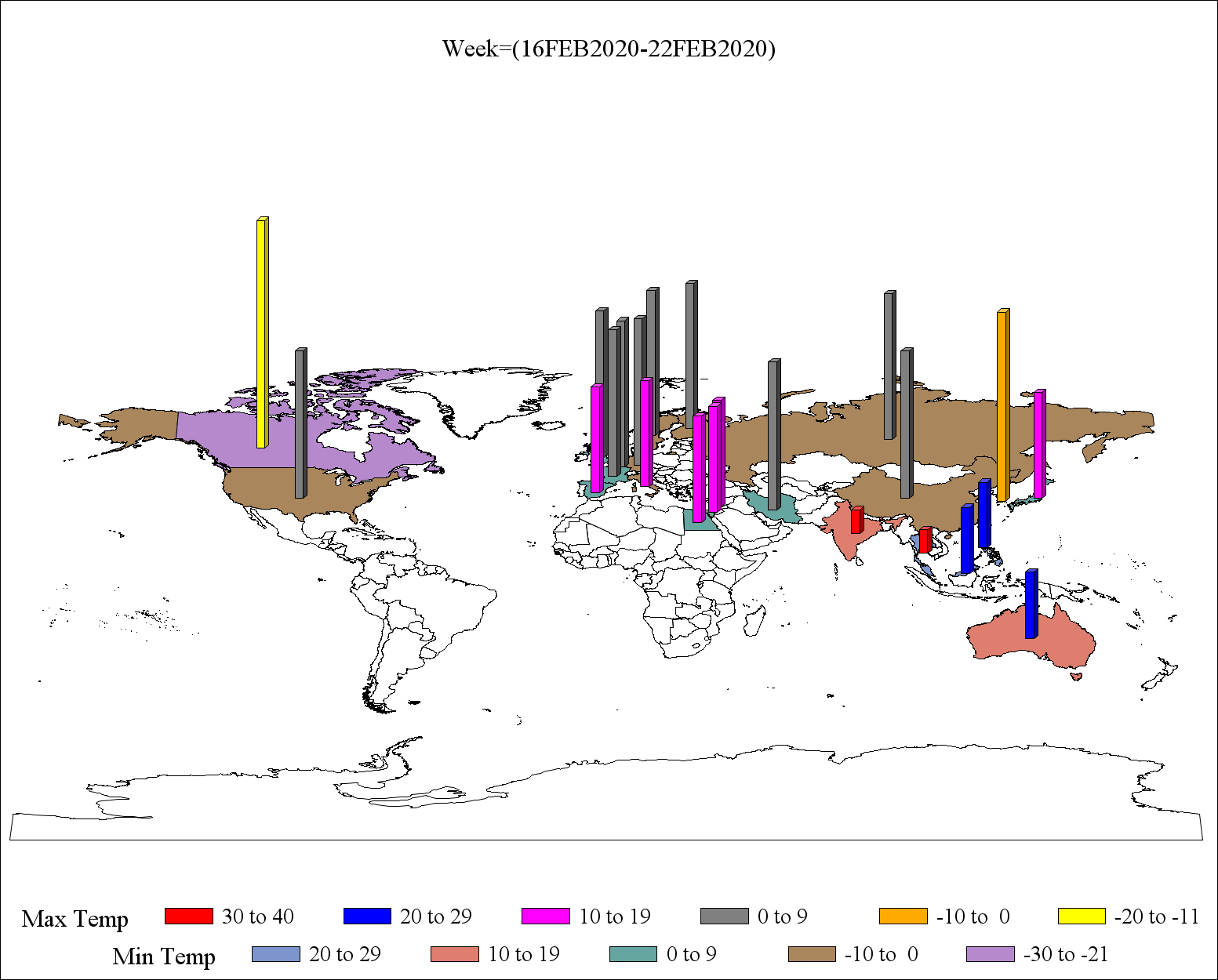


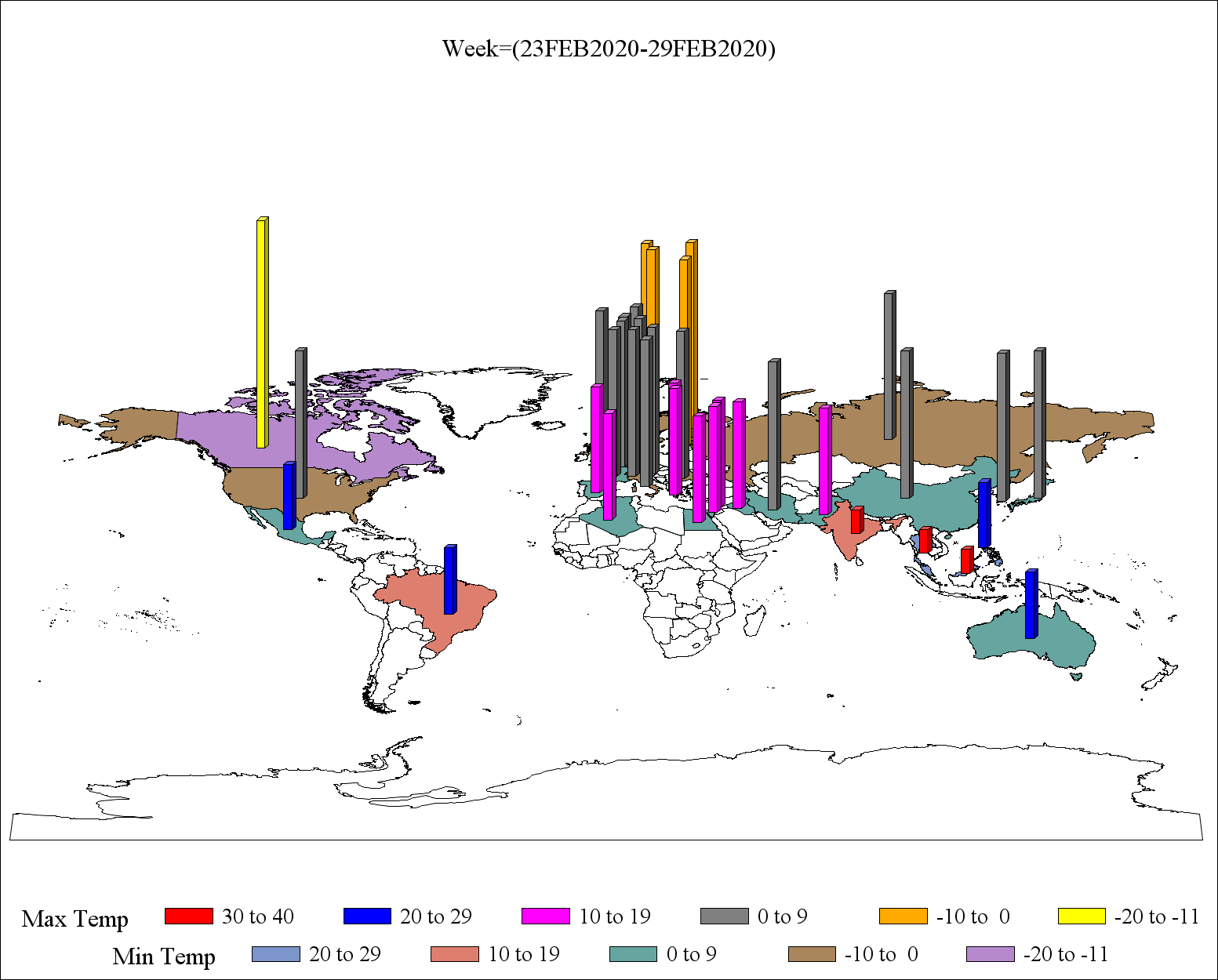


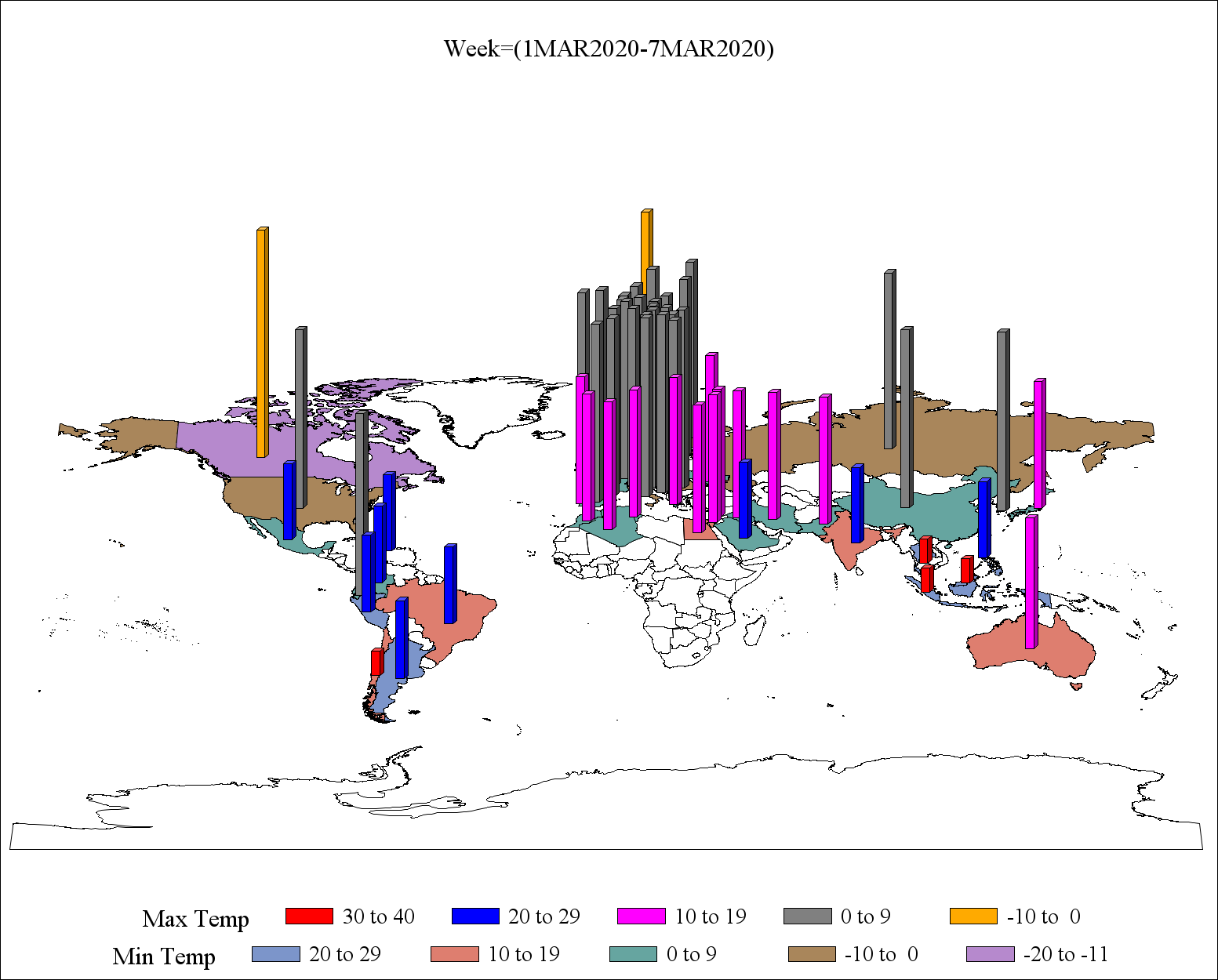


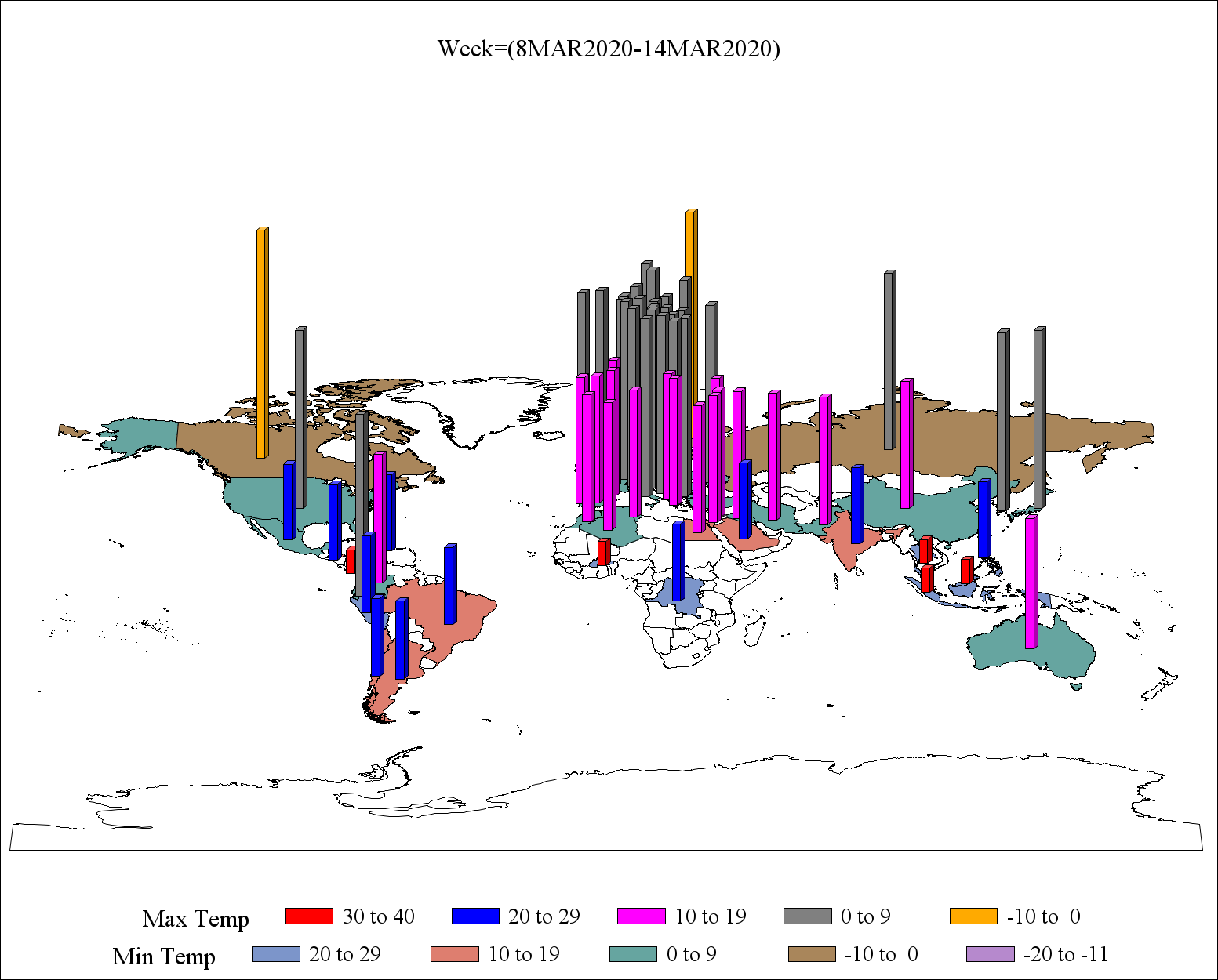


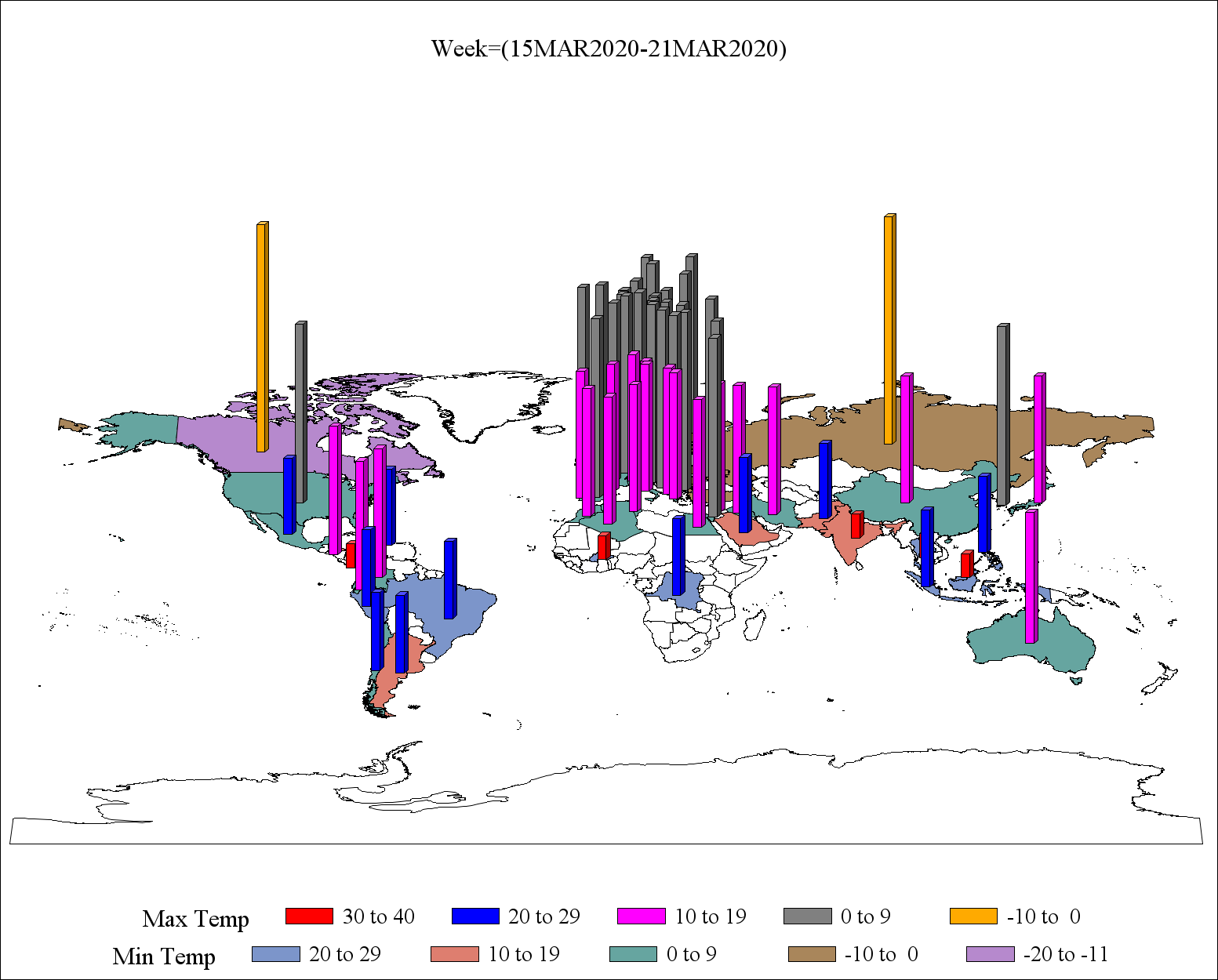


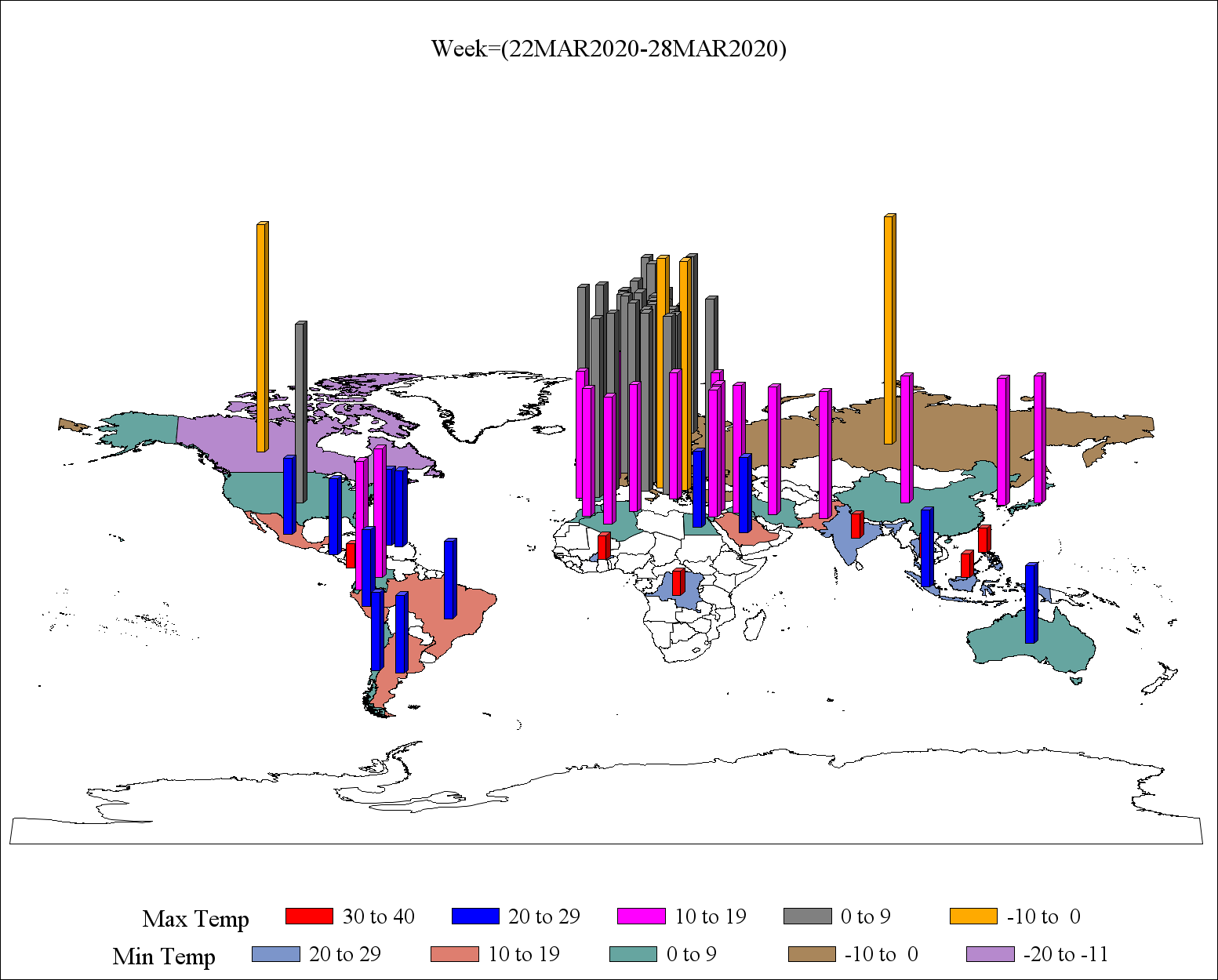


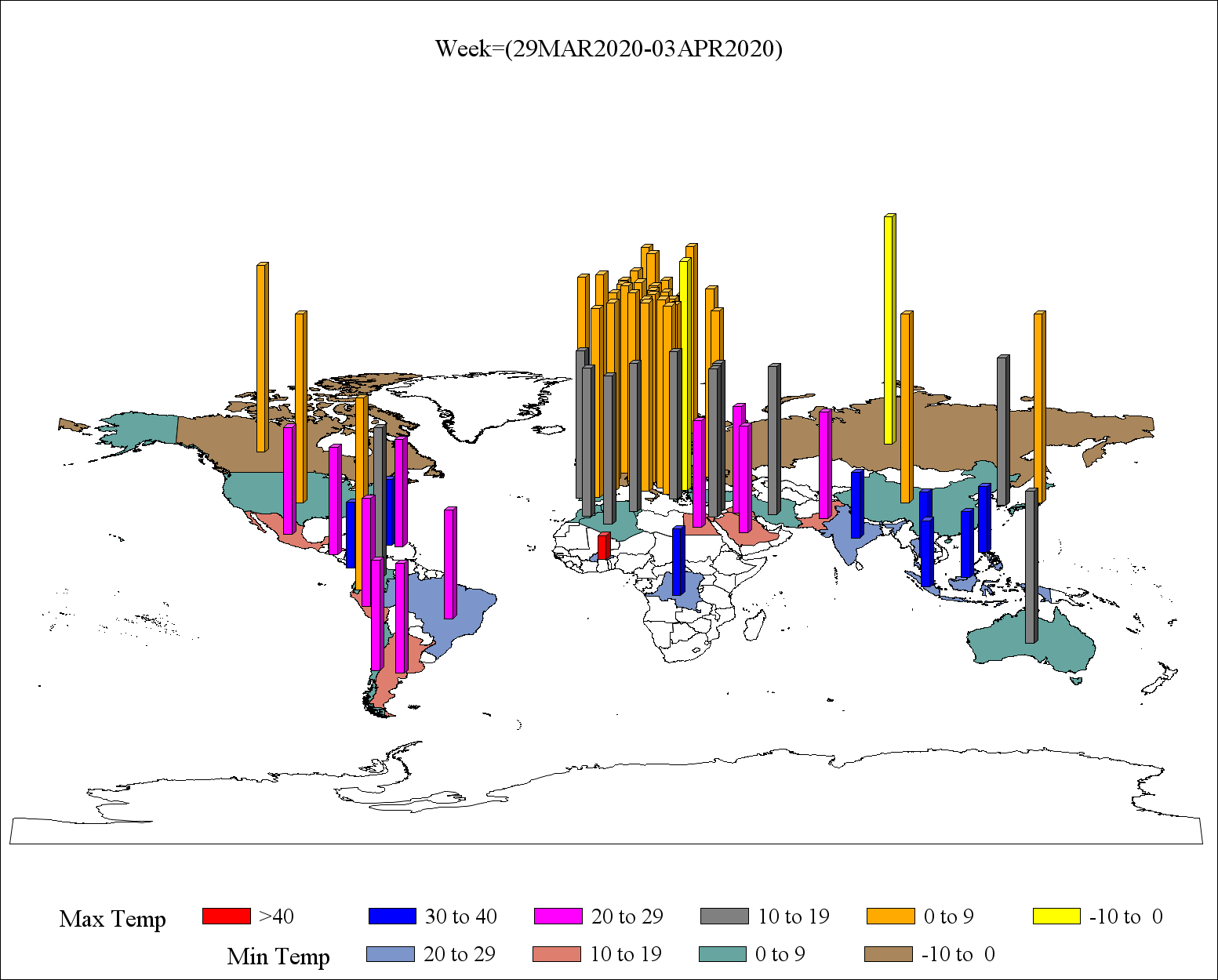

Supplement: S2 Fig — Weekly geographic distribution worldwide: Week 1 (22 JAN 2020–25 JAN 2020), Week 2 (26 JAN 2020–01 FEB 2020), Week 3 (02 FEB 2020–08 FEB 2020), Week 4 (09 FEB 2020–15 FEB 2020), Week 5 (16 FEB 2020–22 FEB 2020), Week 6 (23 FEB 2020–29 FEB 2020), Week 7 (01 MAR 2020–07 MAR 2020), Week 8 (08 MAR 2020–14 MAR 2020), Week 9 (15 MAR 2020–21 MAR 2020), Week 10 (22 MAR 2020–28 MAR 2020), Week 11 (29 MAR 2020–03 APR 2020). (DOC) [file pone.0240710.s002.doc]

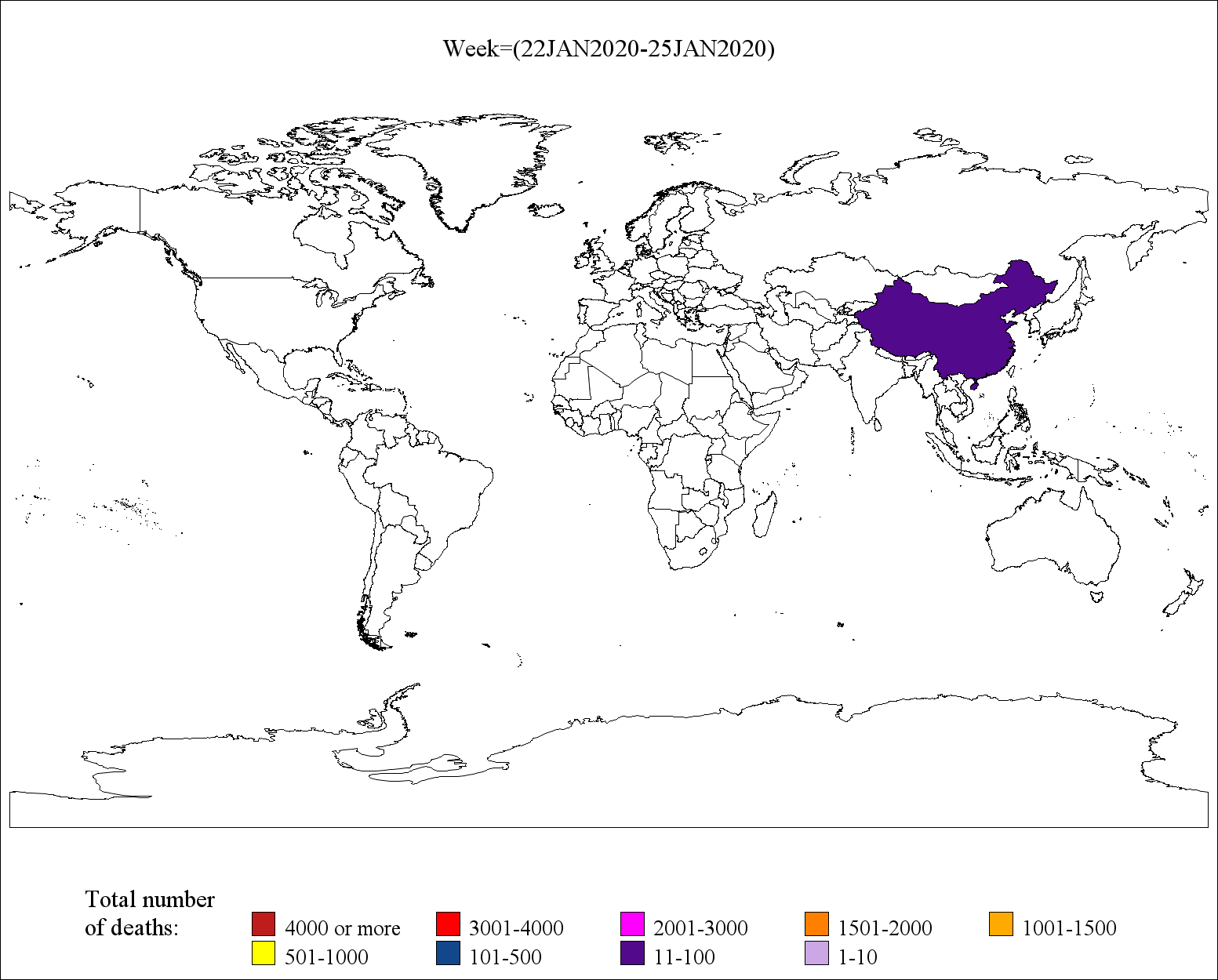


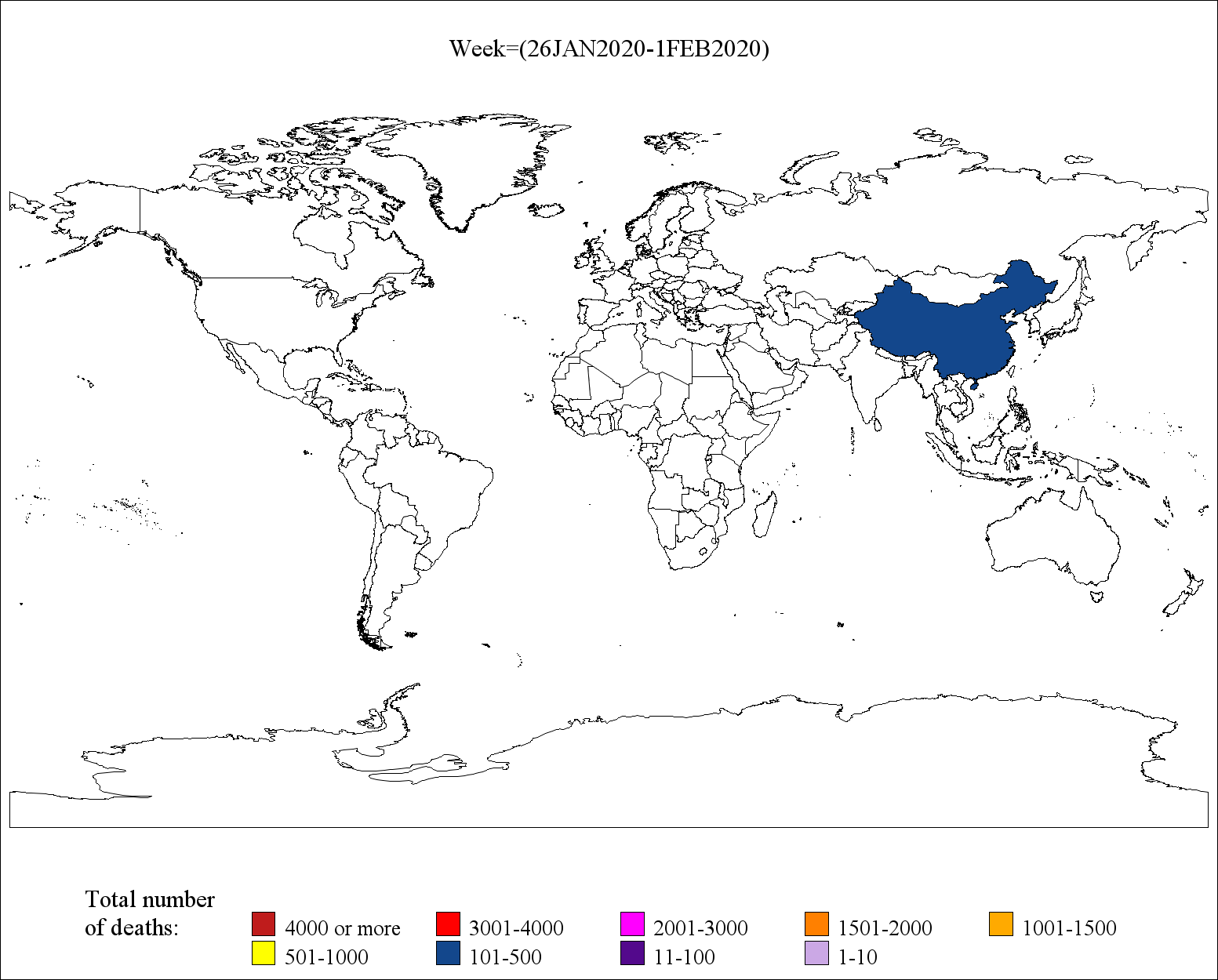


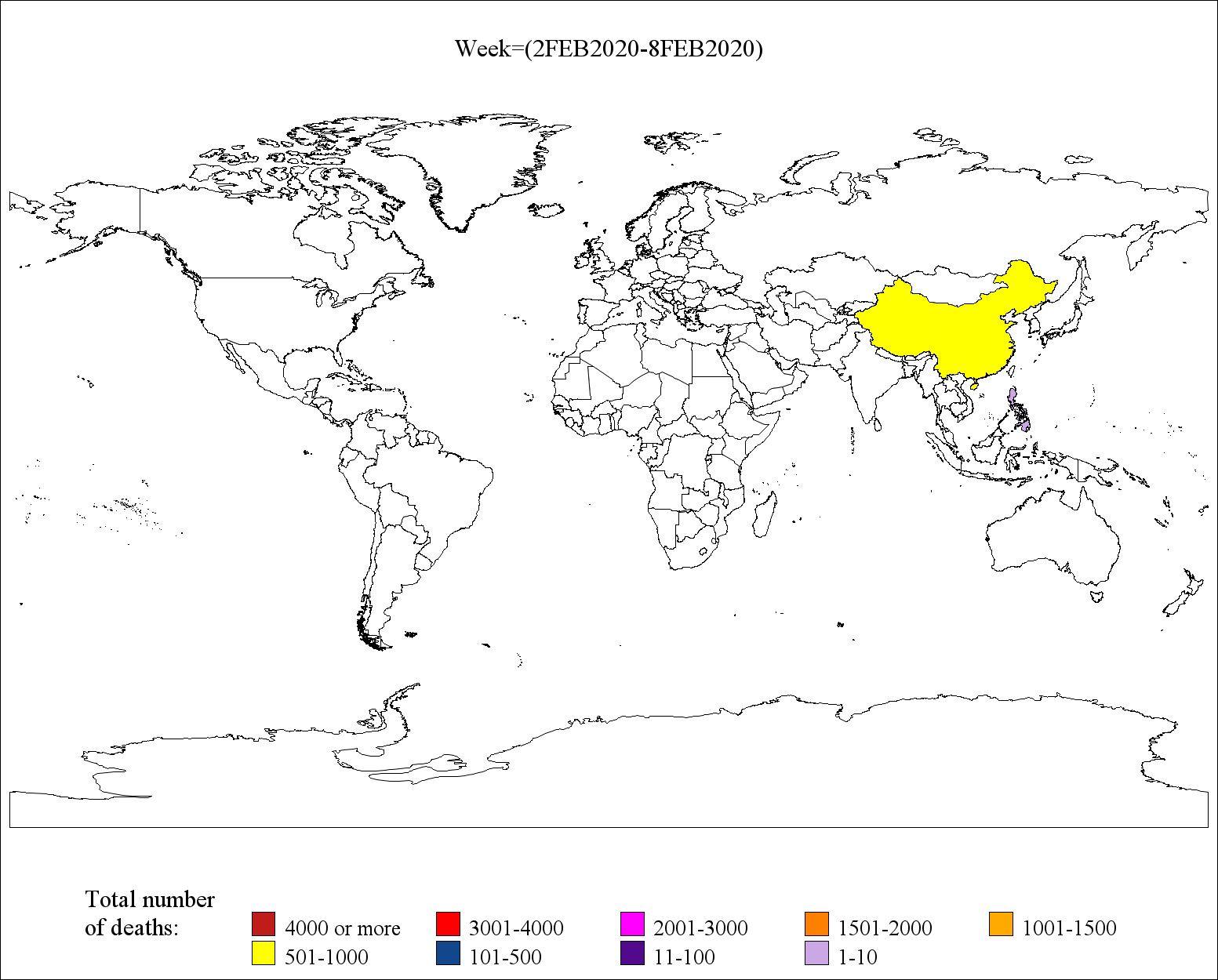


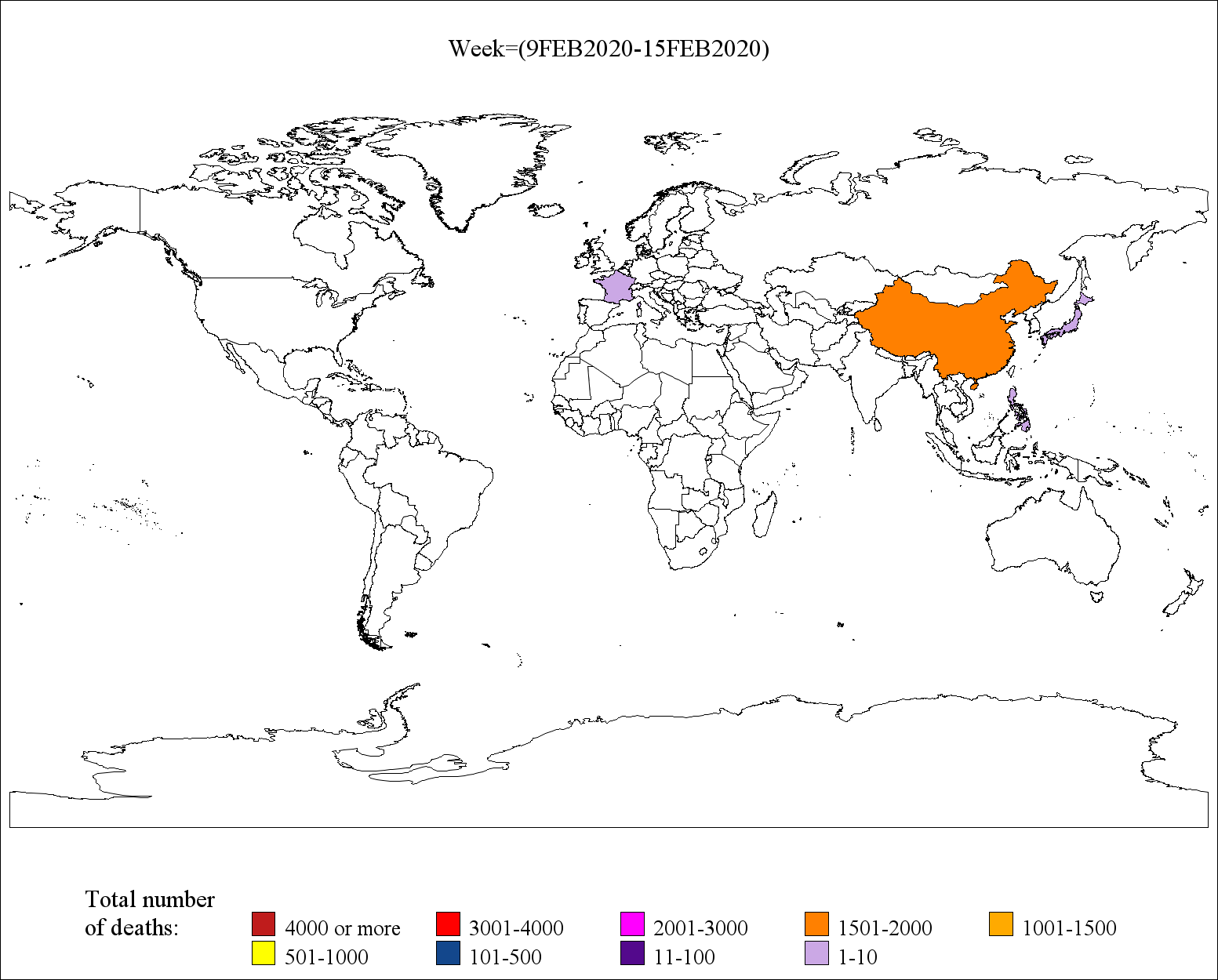


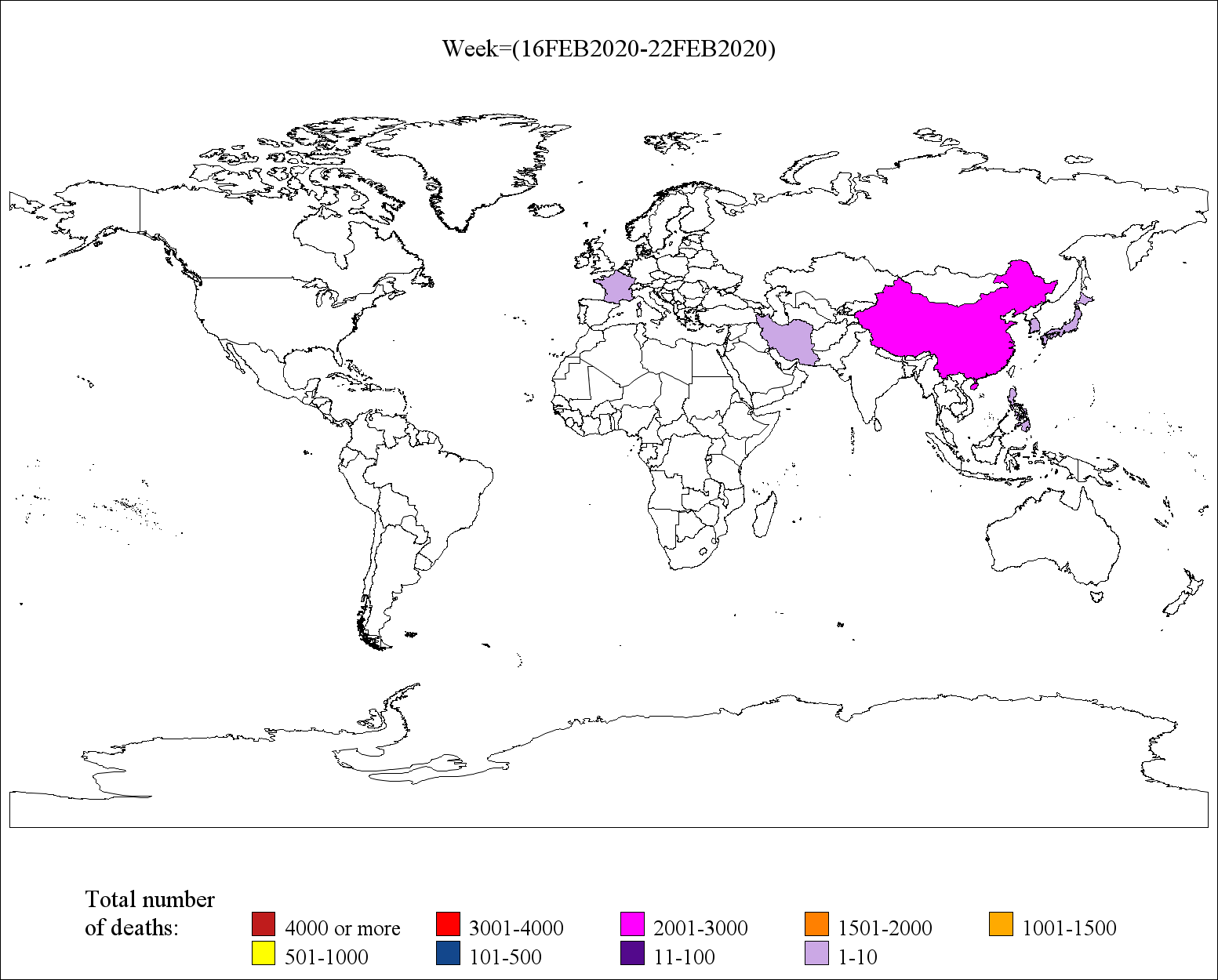


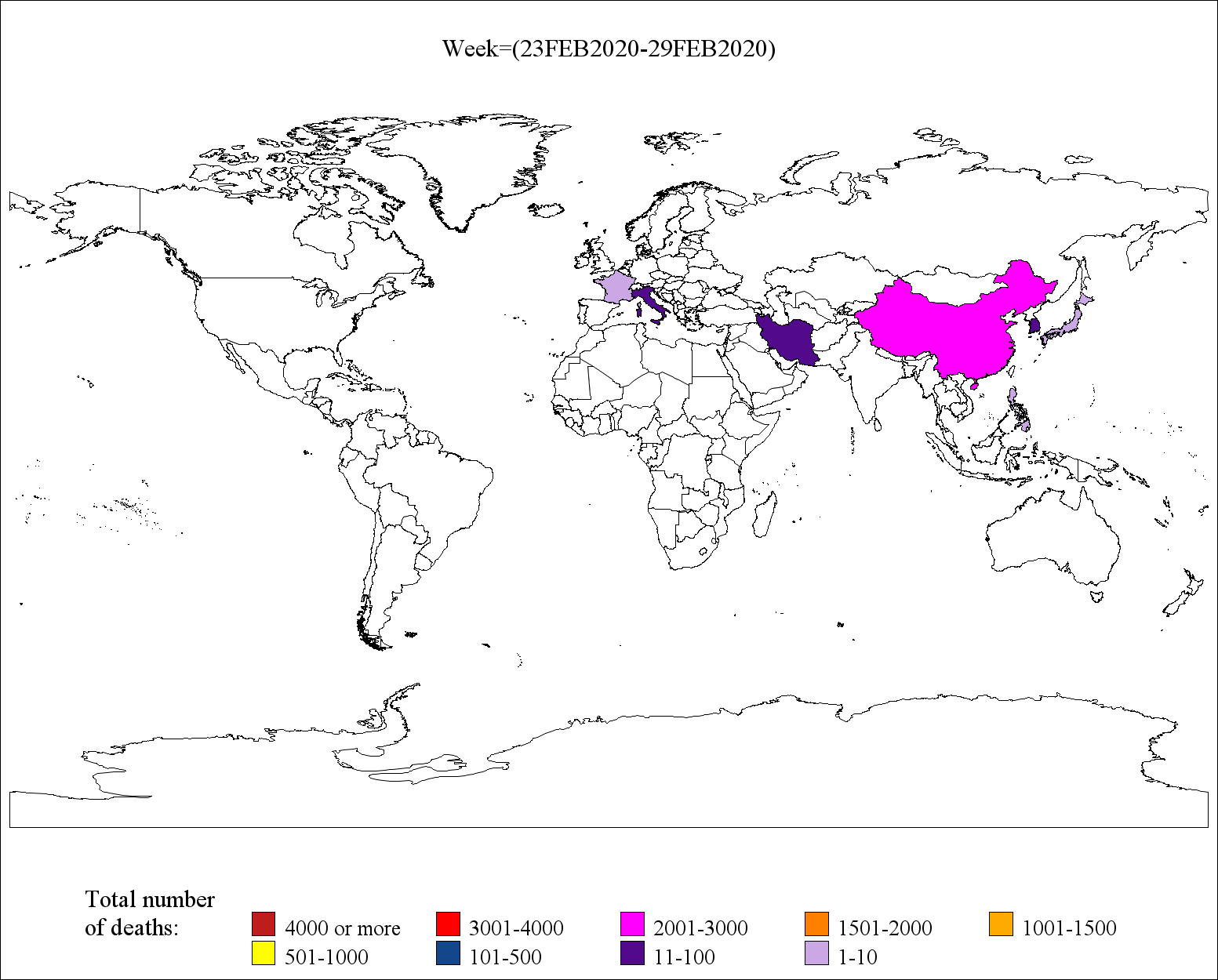


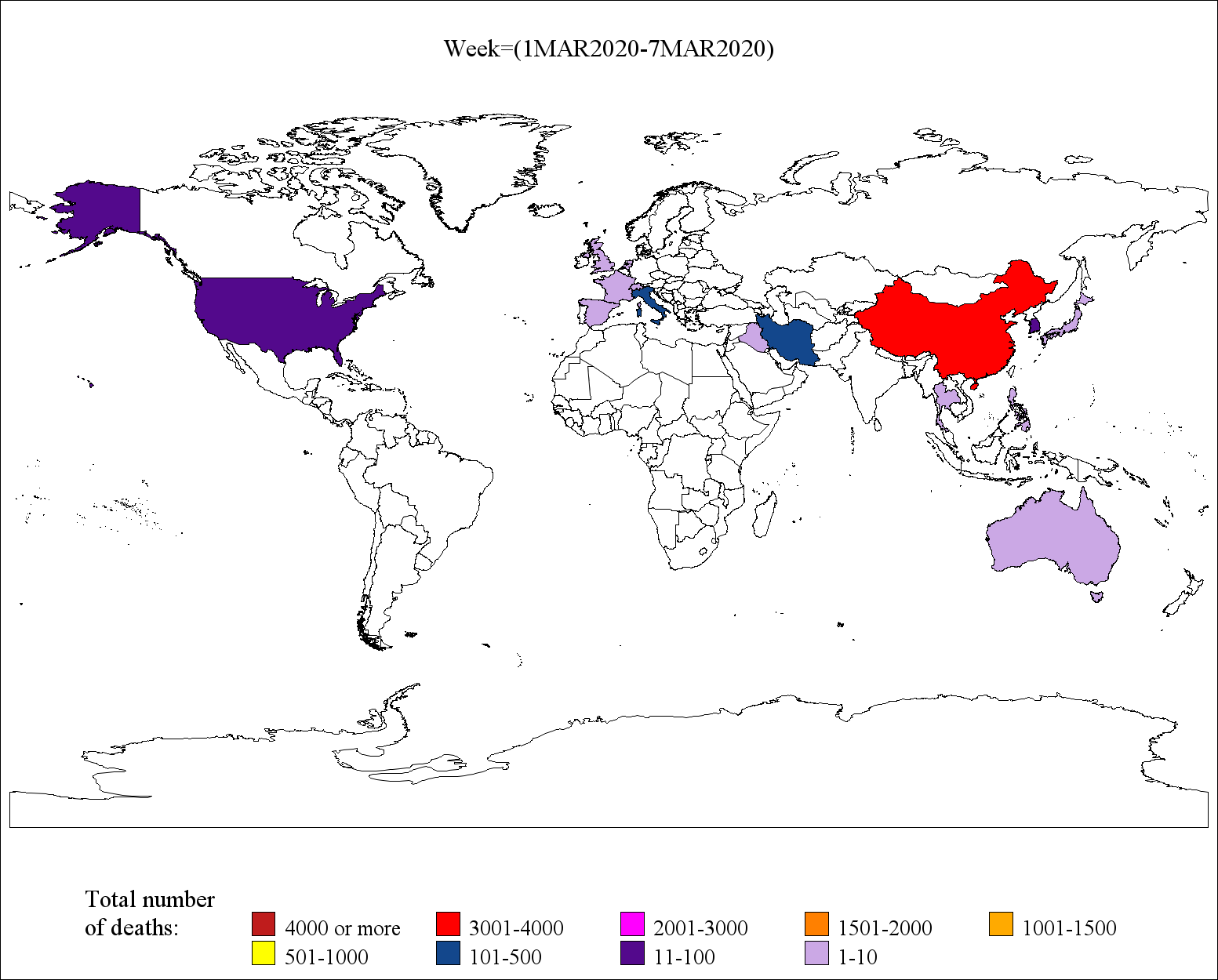


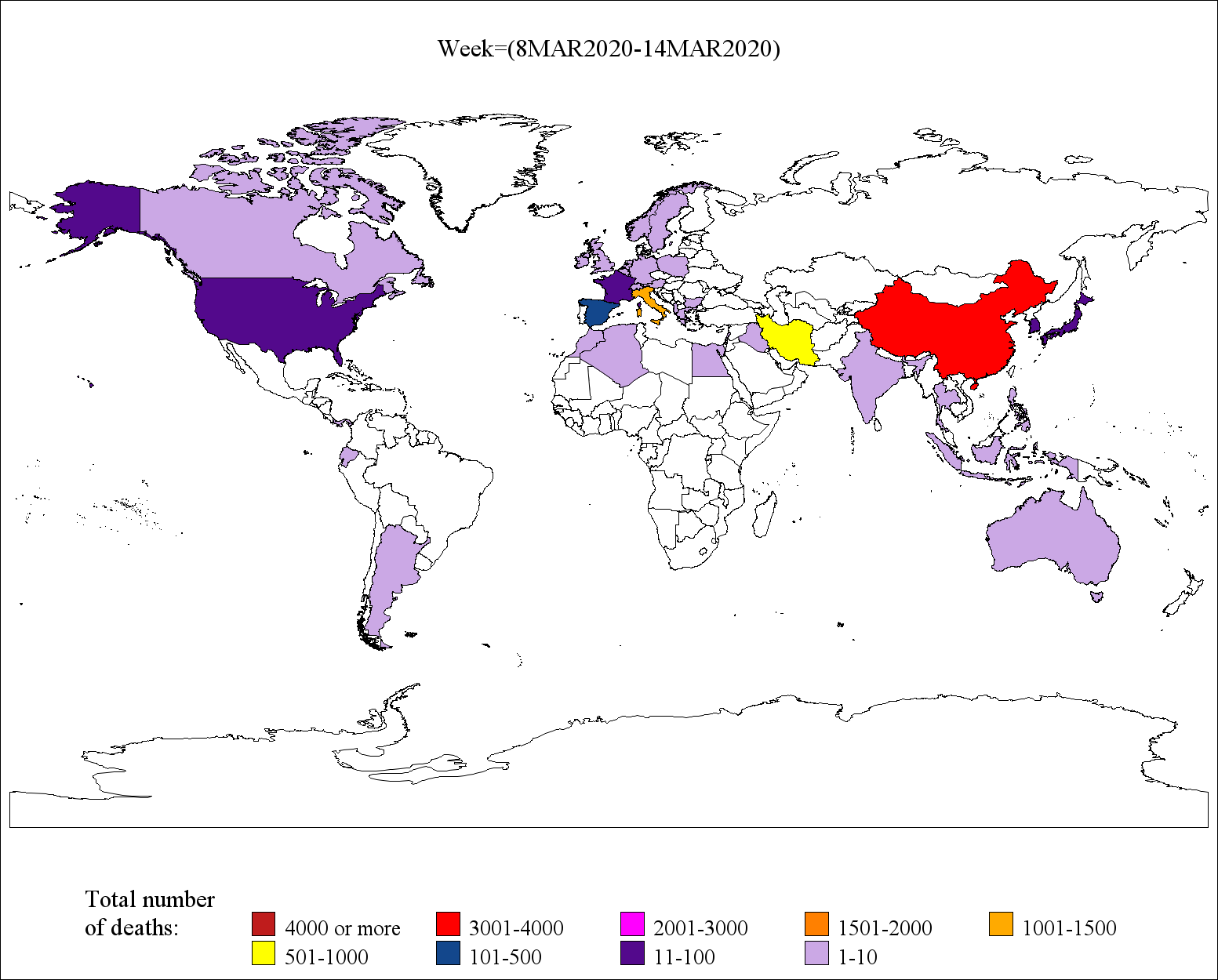


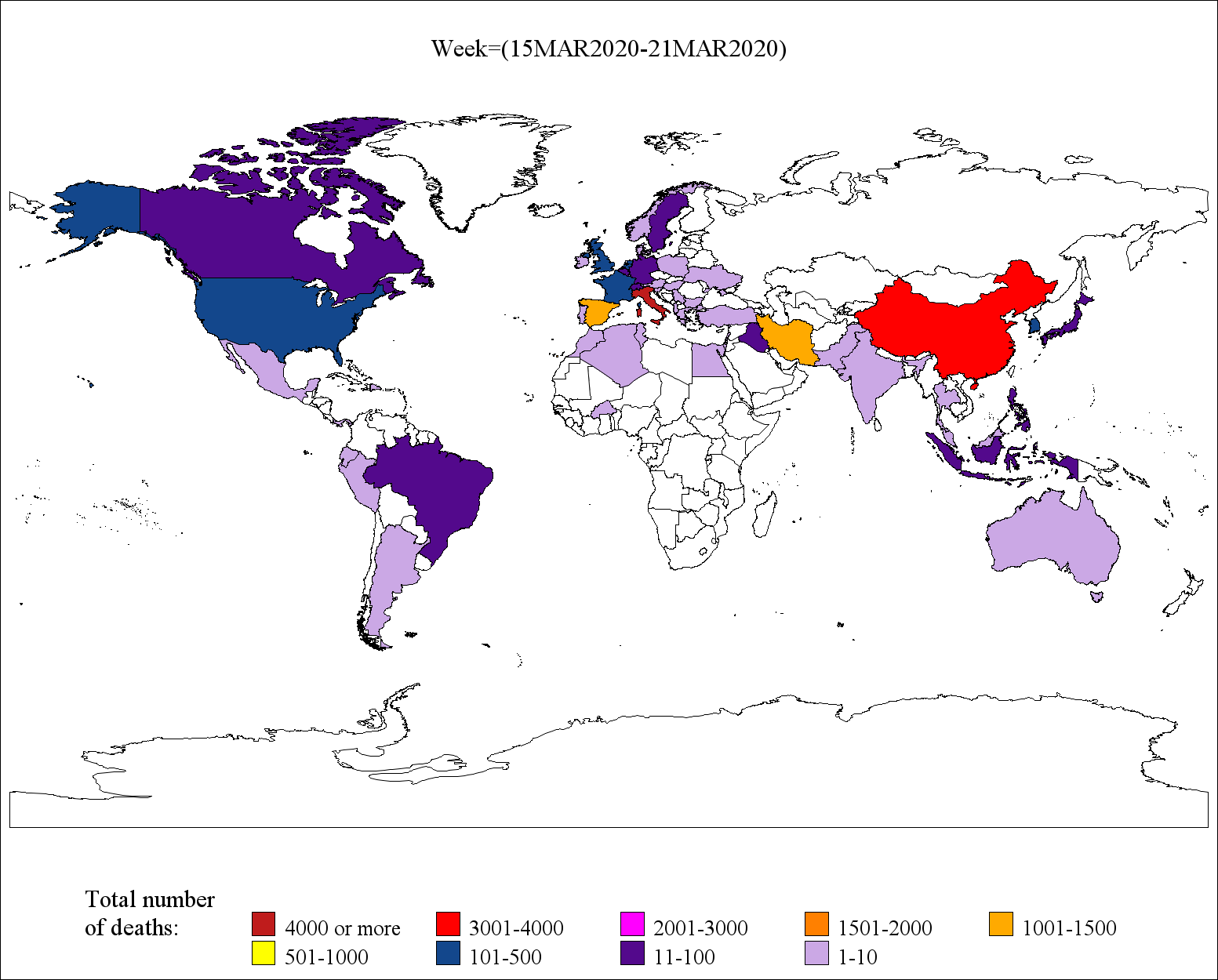


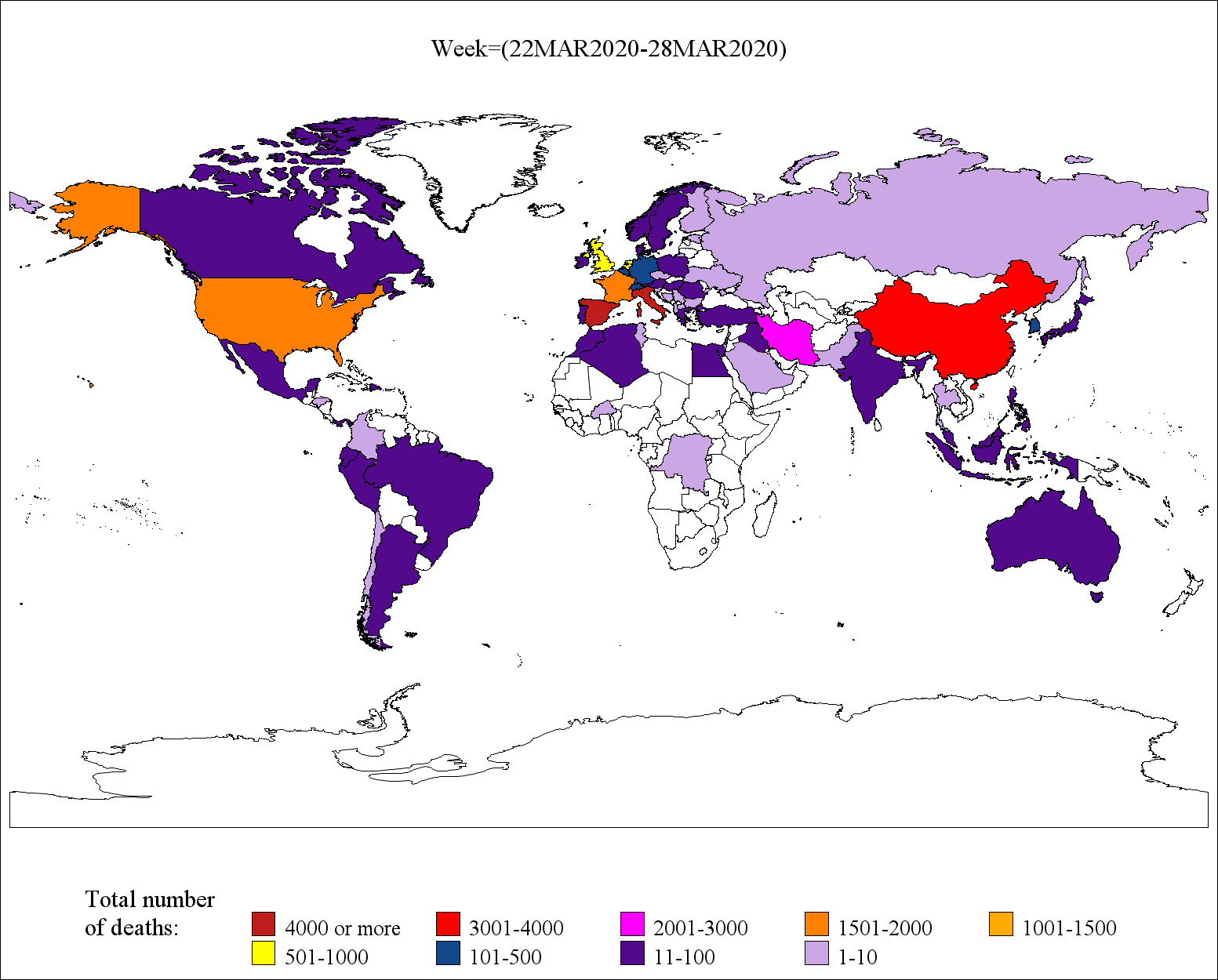


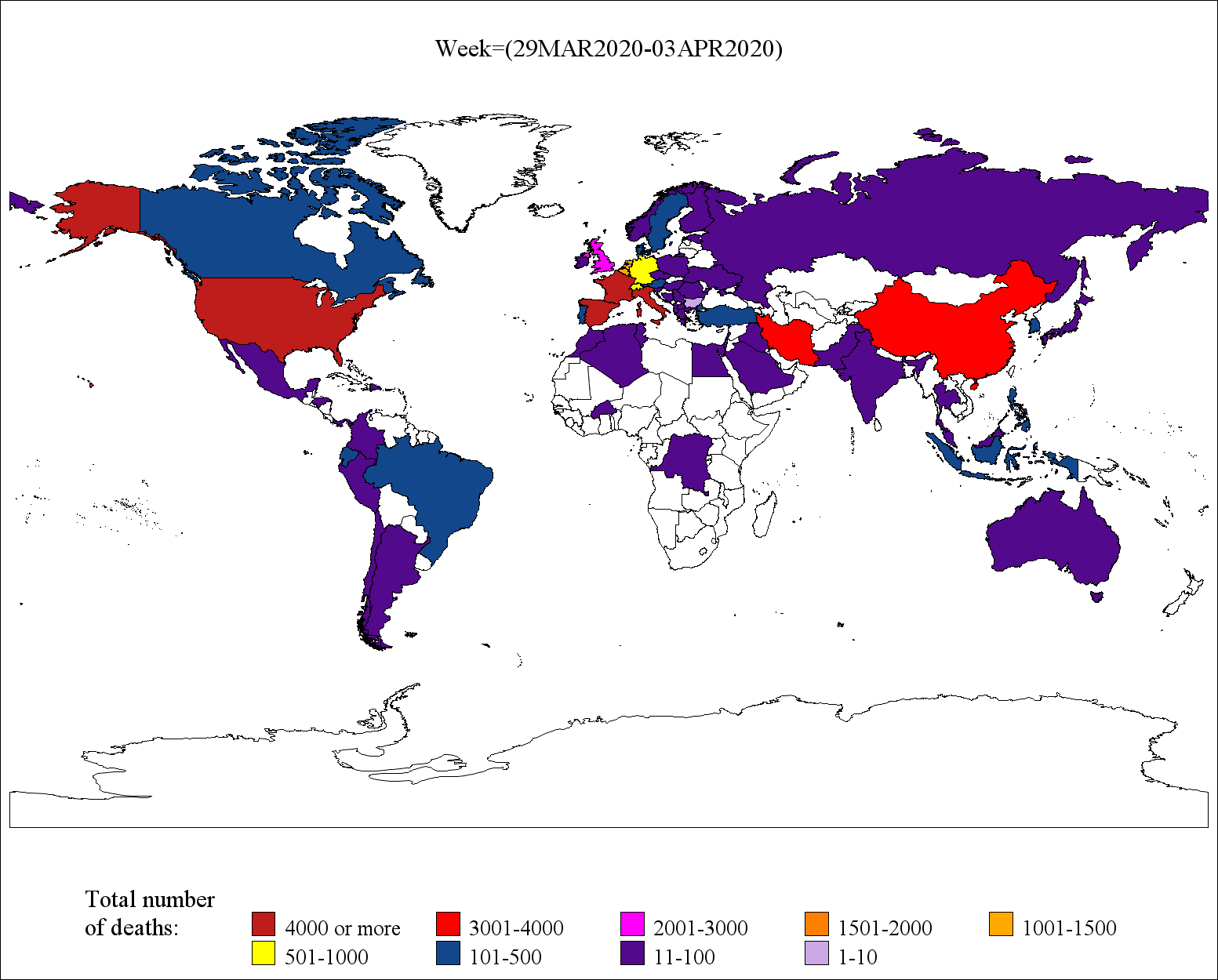

Supplement: S3 Fig — Weekly geographic distribution worldwide: Week 1 (22 JAN 2020–25 JAN 2020), Week 2 (26 JAN 2020–01 FEB 2020), Week 3 (02 FEB 2020–08 FEB 2020), Week 4 (09 FEB 2020–15 FEB 2020), Week 5 (16 FEB 2020–22 FEB 2020), Week 6 (23 FEB 2020–29 FEB 2020), Week 7 (01 MAR 2020–07 MAR 2020), Week 8 (08 MAR 2020–14 MAR 2020), Week 9 (15 MAR 2020–21 MAR 2020), Week 10 (22 MAR 2020–28 MAR 2020), Week 11 (29 MAR 2020–03 APR 2020). (DOC) [file pone.0240710.s003.doc]
