# Supplementary material for: Global impact of environmental temperature and BCG vaccination coverage on the transmissibility and fatality rate of COVID-19
Source: PLoS One. 2020 Oct 22;15(10):e0240710. doi: 10.1371/journal.pone.0240710 (PMC7580966; doi:10.1371/journal.pone.0240710)
Supplement: S1 File — (DOCX) [file pone.0240710.s004.docx]

Under the present study, data on outcome variables (number of new cases and mortality per day) were collected for region-wise worldwide. Here, regions were nested within countries. The variation may prevail at both levels, region level and country level. To quantify variation at each level, multilevel analysis may be applied. For this, based on exploratory analysis, multilevel negative binomial regression model involving 2-level data structure, level-1 (region) and level 2 (country) was considered.

$$\log\left( Y_{\mathrm{ij}} \right)=\beta_{00}+\beta X_{\mathrm{ij}}+\delta W_{j}+u_{j}+e_{\mathrm{ij}}$$

Where,

$$\mu_{j}\sim N\left( 0, \sigma_{\mu}^{2} \right), \& e_{\mathrm{ij}}\sim N(0, \sigma_{\mathrm{ij}}^{2})$$

Where,

Y_ij_ is number of new cases (or mortality) per day in i^th^ region in j^th^ country;

X_ij_ and W_j_ are vectors of region and country level characteristics; and

β and δ are vectors of estimated regression coefficients for the respective covariates.

u_j_: unobserved variation at country level

e_ij_ : error terms at region level.
